# Hybrid physical–statistical framework for seasonal streamflow forecasting in the Upper Feather River Basin, California

**DOI:** 10.1038/s41598-025-15932-7

**Published:** 2025-08-30

**Authors:** Z. Ozcan, Y. Iseri, F. Ulloa, N. Imbulana, E. Snider, M. Mure-Ravaud, M. L. Anderson, M. L. Kavvas

**Affiliations:** 1 Hydrologic Research Laboratory, Department of Civil & Envr. Engineering, University of California, Davis, CA USA; 2 Now at the Arid Land Research Center, International Platform for Dryland Research and Education, Tottori University, Tottori, Japan; 3 California Department of Water Resources, Sacramento, CA USA

**Keywords:** Hydrology, Hydrology, Projection and prediction

## Abstract

Seasonal streamflow forecasts are essential given climate-driven extremes that breach stationarity in traditional methods. The complex hydrology and competing demands necessitate improved forecasting in the Upper Feather River Basin (UFRB), a key California State Water Project source upstream of Oroville Dam. We introduce a hybrid framework combining dynamical downscaling via WRF and the WEHY-HCM snow-hydrology model with a lead-time–dependent exponential-smoothing filter that adaptively corrects bias and quantifies uncertainty. Applied to December–July ensemble forecasts for water year 2024 using hindcast error training (2018–2023), this approach reduced RMSE by 8.7–318.3 million m³ across eight initialization months and eliminated systematic bias. The resulting 10–90% exceedance bands captured ~ 80% of observed flows, offering reliable confidence intervals. This hybrid method delivers accurate, low-bias streamflow forecasts for reservoir operations, flood mitigation, and irrigation planning in the UFRB and provides a transferable template for other basins facing hydroclimatic variability.

**Supplementary Information:**

The online version contains supplementary material available at 10.1038/s41598-025-15932-7.

Seasonal streamflow forecasting is an approach for predicting river water flow over an extended period, up to 6–9 months in advance^1^. This type of forecasting offers significant benefits to various water-related sectors, including agriculture, water resource management, disaster risk reduction, and humanitarian aid^2,3^. Seasonal streamflow forecasts can effectively inform water allocation policies by allowing earlier determination of final allocations to farmers during the irrigation season^4^. Hydropower operators in regions dominated by snowmelt can use seasonal forecasts to plan reservoir drawdown in early winter in anticipation of future inflows^5^. However, the potential benefits of these decisions rely on forecast accuracy, which is becoming more challenging as the frequency/magnitude of floods and droughts increase due to climate change^6–8^.

Various methods exist for seasonal streamflow forecasting. These methods can be broadly categorized into three main groups^9^: (i) Regression-based (Statistical) Approaches; (ii) Ensemble Streamflow Prediction (ESP); and (iii) Forecast-driven Hydrologic Modeling. Regression-based approaches are entirely data-driven models that directly connect past snow, antecedent flows, climate indices, etc., to seasonal streamflow^10–12^. ESPs involve the application of a physically based hydrological model run with ensembles of historical meteorological inputs. Forecast skill is determined entirely by the accurate initialization of the system, specifically the initial conditions^13^. Like ESPs, Forecast-driven Hydrologic Modeling uses the physically based hydrological models, but now the models are forced with seasonal meteorological forecasts (e.g., from GCMs or RCMs)^14–16^.

Traditional regression-based or statistical forecasting methods have proven insufficient in capturing the complexities of extreme events, highlighting the need for more robust, physically based approaches^17–19^. The primary motivation for ESP-based methods is the unsuitability of regression-based approaches in the context of nonstationarities associated with climate change and variability^20^. Since ESPs rely solely on historical weather data, they assume a stationary climate regime. As a result, in a non-stationary world^21^ESP forecasts may be biased and may understate emerging extremes^22^. Thus, forecast‑driven hydrologic modeling offers a more robust framework than purely statistical or ESP‑based methods—by combining accurate initial‑condition memory with skillful seasonal meteorological forecasts, it overcomes the stationarity and input‑only limitations of those approaches. However, its performance still hinges on the quality of the climate forecasts and the hydrologic model’s parameterizations.

Despite the advances of forecast-driven systems, to our knowledge, few studies, such as that by Xiao et al.^23^have applied a combined deterministic–statistical seasonal forecasting framework specifically focused on the Upper Feather River Basin (UFRB), a major source of California’s water needs that provides almost all of the water delivered by the California State Water Project^24^. The UFRB is located high in the northern Sierra Nevada and feeds into the North, Middle, South, and West Forks of the Feather River, just upstream of the Oroville Dam. It serves as a vital component of the California State Water Project, providing essential water supplies for urban, agricultural, and industrial needs throughout the state. The basin has a typical Mediterranean climate, characterized by wet, snowy winters that transition into hot, dry summers. As a result, much of its annual runoff comes from snowmelt. However, occasional rain-on-snow events can lead to large runoff pulses and increased flood risks in the spring. During the dry season, it is essential to maintain base flows for downstream requirements. These conditions create a constant operational challenge: managing the water stored behind Oroville Dam to ensure there is enough to support low flows during the summer, while also releasing water early enough to reduce flood risks as snowmelt accelerates^24,25^.

In this study, we combine a physics-based deterministic ensemble forecasting system with a straightforward yet effective statistical correction method. First, we create raw seasonal inflow forecasts by downscaling global climate forecasts from the CFSv2 using the Weather Research and Forecasting (WRF) model. This generates high-resolution meteorological data for the UFRB. These meteorological fields then drive the WEHY-HCM hydrologic model, which simulates snow accumulation, melt, and basin inflows based on the predicted weather conditions. To address systematic biases present in both the climate forecasts and hydrologic parameters, we implement a seasonal, lead-time-dependent exponential-smoothing (SES) algorithm on the deterministic forecast errors at each lead time. This approach produces bias-corrected inflow predictions. The methodology was applied to forecast seasonal inflows to Oroville Dam from December 2023 to July 2024.

## Results

This section presents the deterministic ensemble forecast results for the 2024 water year, alongside hindcasting data from 2018 to 2023 used to develop the SES model (see Supplementary Information Figs. S1-S6). The SES model’s application to the 2024 water year and the results are discussed in the SES Results section, with monthly streamflow forecasts compared to FNF (Full Natural Flow) data at the FTO station for evaluation.

### Deterministic forecasting results

**Fig. 1:**
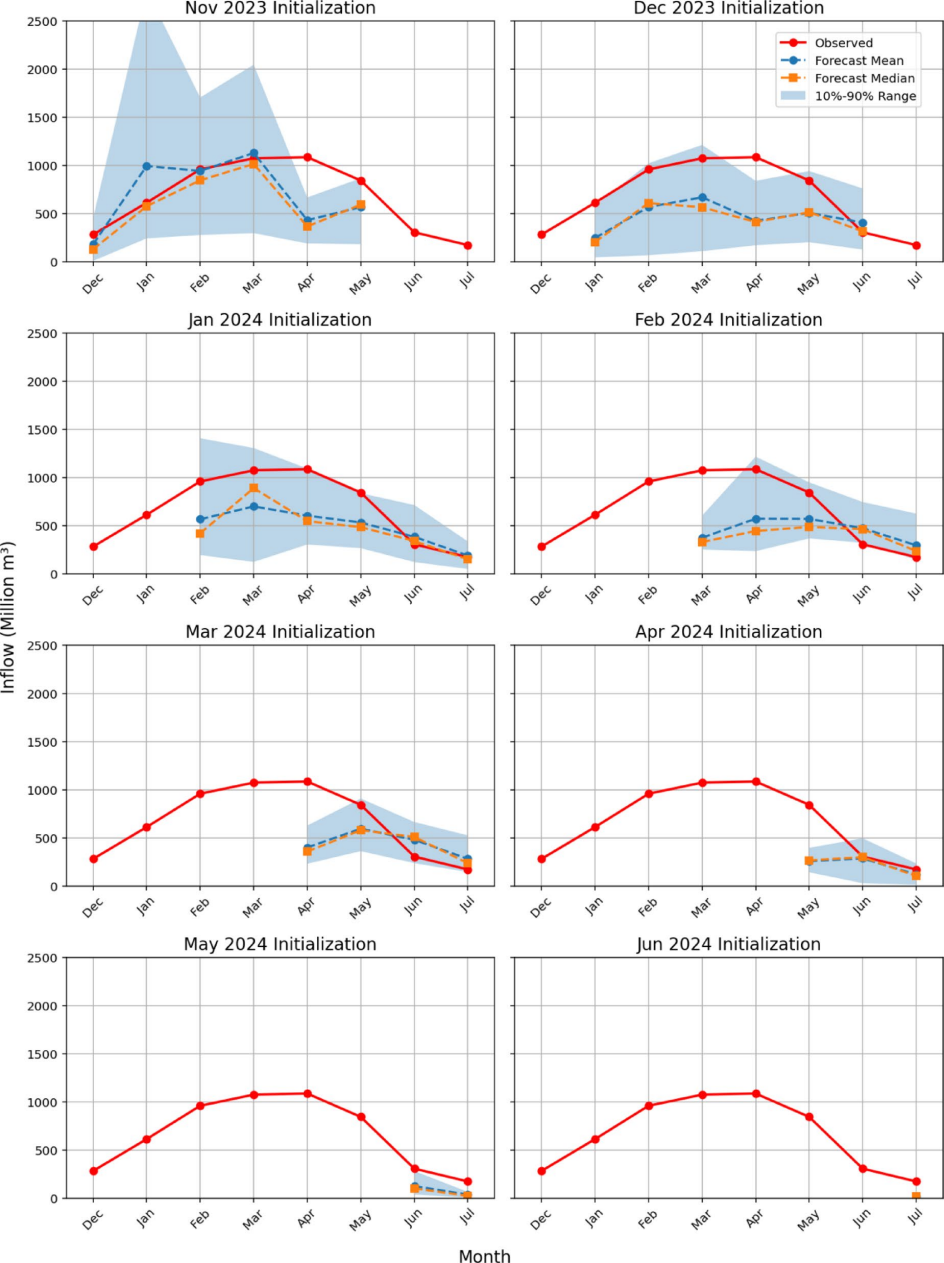
Seasonal streamflow forecasts to the FTO station for water year 2024 in the UFRB. Each subplot corresponds to a different forecast initialization month (November–December 2023; January–June 2024) and displays observed inflows from December 2023 through July 2024 (red circles), forecast mean (blue circles), forecast median (orange squares), and the 10–90% exceedance range (shaded band). The common x-axis spans December through July, and the y-axis shows inflow in million m³.

Figure 1 depicts the deterministic flow forecasts at the UFRB watershed outlet for the 2024 water year, based on 15-member ensembles initialized monthly from November 2023 to June 2024. It includes the ensemble mean, median, and 10% and 90% exceedance probabilities compared to FNF data at the FTO station. Because CFSv2 provides up to six months of forecast lead time, each monthly initialization yields a varying forecast horizon (Supplementary Table S1): the November and December initializations cover ahead through May and June, respectively, while January through June initializations extend through July. Supplementary Table S2 provides RMSE and PBIAS for each initialization month, and the following paragraphs discuss the performance of the forecast system for each month.

- *The November initialization* typically underestimates actual flows, except for January and March. The most significant discrepancy occurs in April. The FNF values are generally captured within the 10%−90% exceedance range, represented by the shaded area, except for the FNF value in April.
- Similarly, *the December initialization* tends to underestimate streamflow as well. Again, the only month not included within the exceedance range is April.
- The *January initialization* underestimates inflows from February through June, with observed values consistently above the forecast mean and median. The gap narrows from April onward, and by June the forecasts almost match observations. Early-season uncertainty is highest in February–March, as shown by the widest 10–90% exceedance bands, then steadily decreases into June–July, reflecting growing confidence. That observed values remain within the uncertainty band throughout confirms the ensemble’s ability to capture inflow variability despite central biases.
- *The February initialization* reveals that the model tends to underestimate inflows during the early months of the forecast period, particularly in March and April. From May onwards, the forecast median and mean begin to align more closely with the observed values, particularly in June and July, indicating improved accuracy later in the season. The 10–90% exceedance range is the widest in April, indicating a higher degree of uncertainty in this month. Additionally, the observed values are encompassed by the 10–90% exceedance range in all months except March and June.
- *The March initialization* indicates that the ensemble mean and median underestimate the observed inflows in April and May, while they overestimate the inflows in June and July. However, the extent of overestimation in June and July is less pronounced compared to the underestimation in the earlier months. The width of the 10–90% exceedance range is relatively stable during the forecast period, suggesting a similar uncertainty level in all months.
- *The April initialization* results in nearly perfect forecasts for June and July but significantly underestimates the inflow in May. During May, the observed inflow exceeds even the 10–90% exceedance range, indicating that the model fails to capture the magnitude of the observed inflow.
- *The May initialization* underestimates the inflows for June and July, with the observed inflows falling outside the 10–90% exceedance range. This indicates that the model fails to adequately capture the actual inflow values during these months, underestimating their magnitude.
- Lastly, *the June initialization*, which has a monthly inflow forecast only for July, underestimates the observed inflow.

Based on the analysis of all initializations from November to June, the model shows mixed performance across different months and flow conditions. The model tends to underestimate flows during peak periods, particularly in March, April, and May, where the observed values often fall outside or exceed the forecast range, including the 10–90% exceedance band. This suggests that the model struggles with capturing higher flow events accurately, indicating a bias toward lower estimates during periods of increased inflow. However, the model’s performance improves considerably during lower flow periods, as seen in the closer alignment of forecast mean and median values with observed inflows in later months, such as June and July, for some initializations. The uncertainty bands also tend to narrow toward the later part of the season, indicating increased consistency and confidence in the forecasts during periods of lower variability. Overall, while the model can effectively capture seasonal trends and performs well during periods of relatively stable or low inflows, improvements are needed in its ability to predict higher flows and encompass the variability during peak months.

### Seasonal exponential smoothing (SES) results

**Fig. 2:**
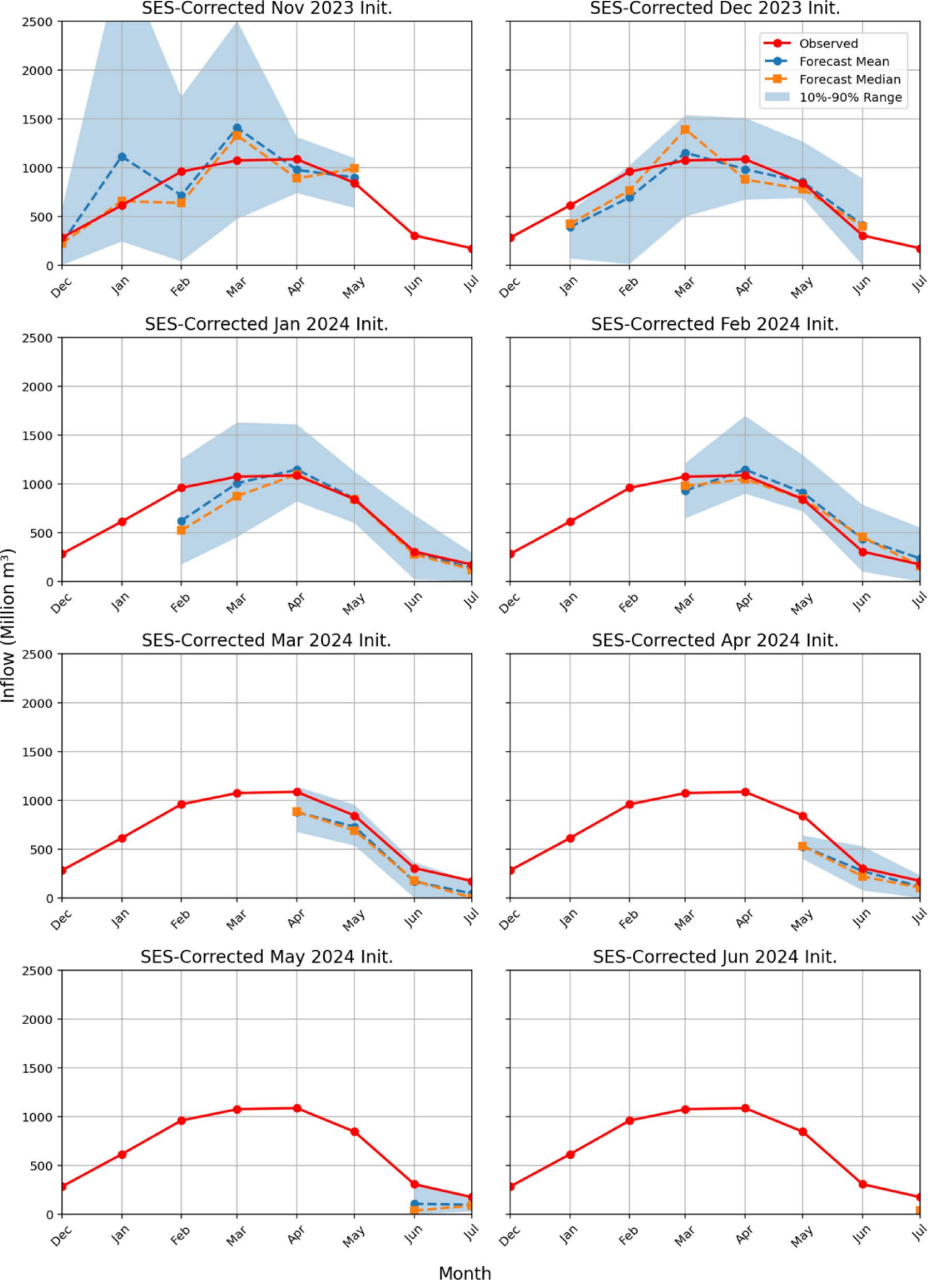
Seasonal streamflow forecasts for the FTO station in water year 2024, corrected using SES, are displayed by forecast initialization month (Nov 2023–Jun 2024). Each subplot shows observed inflows (red circles), forecast mean (blue circles), median (orange squares), and the 10–90% exceedance range (shaded band) from Dec 2023 to Jul 2024, with the x-axis spanning these months and the y-axis in million m³.

Figure 2 illustrates the corrected streamflow forecasts at the FTO station at the watershed outlet of the UFRB for the 2024 water year, generated using SES. Supplementary Table S3 reports the RMSE and PBIAS for each forecast initialization month. In the following paragraphs, we discuss the performance of the SES-corrected forecast system for each initialization month and compare it to the original deterministic forecast system.

- In *the November initialization*, the FNF values closely align with the corrected streamflow values for December, April, and May. This indicates an improvement over the original deterministic model, which tended to underestimate streamflow during these months. However, the forecast mean and median still struggle to accurately capture streamflow values in January, February, and March. On a positive note, all FNF values are now included within the exceedance range.
- The SES-corrected *December initialization* shows significant improvement compared to the original deterministic model. The SES-corrected means of monthly forecasts are pulled up, much closer to the FNF values. The exceedance range comprises almost all the observed values, except January.
- In the January initialization, SES-corrected streamflow forecasts show significant improvements over the original deterministic forecasts, aligning more closely with observed inflows during March, April, and May. This correction reduces the underestimation bias of the deterministic model. The 10–90% exceedance range of the corrected forecasts captures FNF values more accurately, with observed flows in April and May now fitting within this range. The forecasts for June and July remain similar to the original forecasts, as the deterministic model was already accurate during those lower flow periods.
- The use of SES in the February initialization notably enhances model accuracy, especially during high-inflow months like March, April, and May. SES correction reduces forecast bias and better aligns with observed values while capturing variability in inflows. This improved performance demonstrates SES’s effectiveness in countering underestimation in original forecasts, particularly during higher flow periods, while still maintaining reliability in lower flow months like June and July, showcasing the robustness of the SES-corrected approach.
- The corrected forecasts for *the March initialization* using SES show significant improvement in model performance, particularly in April, where the deterministic model previously exhibited substantial underestimation. The SES correction reduces forecast bias, and the uncertainty band helps better capture the range of potential inflows. In June and July, the corrected forecasts align well with the observed values, maintaining consistency and demonstrating the reliability of SES for stable flow periods.
- The SES-corrected forecasts from *the April initialization* demonstrate notable enhancements, particularly in reducing the underestimation noted in May. Although the uncertainty band does not capture the observed flow in May, the upward adjustment of the forecasted mean and median values reduces the gap between the forecast and observed flows. These improvements highlight the SES model’s ability to address the limitations of the deterministic forecast, especially during peak inflow periods.
- *The May Initialization* SES-corrected forecast shows minor improvements compared to the deterministic forecast, with the forecast mean and median in June and July slightly closer to the observed values. Importantly, the observed values for June and July are now encompassed within the 10–90% exceedance range.
- For *the June initialization*, the SES-corrected forecast shows only marginal improvement over the deterministic forecast. The forecast mean and median still underestimate the observed value in July.

**Fig. 3:**
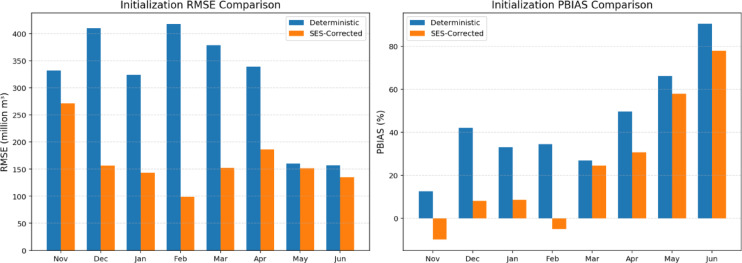
Comparison of the forecast performance by the initialization month (Nov 2023–Jun 2024). Left panel shows RMSE (million m³) for the original deterministic forecasts (blue) versus the SES-corrected forecasts (orange); right panel shows percent bias (PBIAS %) for the same two methods. Positive PBIAS indicates underestimation, negative indicates overestimation.

Figure 3 compares the RMSE and PBIAS values by initialization month for the original deterministic model and the SES-corrected forecasts. The visual interpretations made through Fig. 2 are also supported by the change in the RMSE and PBIAS values. Both the RMSE and absolute PBIAS values decrease after applying the SES correction, which highlights an improvement in overall forecast performance; however, the degree of improvement varies by the initialization month. The significant improvements in forecast performance are seen in December, January, March, and April initializations.

In summary, the SES-corrected forecasts consistently capture both the timing and magnitude of the seasonal streamflow peak, with forecast means and medians rising and falling in close agreement with observed flows. Systematic bias is minimal—predicted values typically lie within ± 10% of observations—and the 10–90% exceedance bands reliably encompass the actual data points. Uncertainty is greatest for the earliest (November) initialization and steadily narrows for later runs, yet even the longest‐lead forecasts remain skillful throughout the December–July period. Overall, the SES‐corrected system delivers accurate, low‐bias inflow predictions with reliable confidence intervals, making it a robust tool for seasonal water‐resource planning.

## Discussion

In this study, the WRF regional atmospheric model and the WEHY-HCM watershed hydro-climate model were coupled with a lead-time–dependent SES filter to forecast monthly flows at the UFRB outlet during the December–July period of water year 2024. The primary objective of this study was to establish a seasonal streamflow forecasting system by integrating a hybrid physical–statistical framework that enhances forecast accuracy, mitigates systematic bias, and delivers realistic uncertainty estimates beyond raw deterministic outputs.

Application results demonstrate that the SES-corrected forecasts consistently outperform the uncorrected deterministic runs in two key metrics: RMSE and PBIAS. Across all eight initialization months, RMSE declined by 8.6–318.2 million m³, with the largest reductions for early-winter through spring initializations (December – April). Similarly, PBIAS values moved much closer to zero after SES correction, indicating that systematic over- or under-prediction was substantially mitigated. These improvements confirm that applying an adaptive smoothing of historical error patterns can correct persistent physical-model biases.

The seasonal exponential-smoothing filter delivers these gains by giving greater weight to more recent errors compared to older ones at each lead time. Early-season errors, often driven by uncertainties in snowpack and soil-moisture initialization, are mitigated by the SES filter’s use of the same-month errors from previous years, which corrects for systematic seasonal discrepancies. For later initializations, when physical-model inputs are already more accurate, the SES filter produces only modest corrections. This lead-time dependence explains both the large error reductions at short leads and the progressively smaller adjustments for May–June forecasts. Equally important, the 10% – 90% exceedance bands produced by the SES filter were broadly reliable. Approximately 80% of the observed inflows fell within these intervals, indicating that the stated confidence levels align well with empirical coverage. Uncertainty is widest for the November run, when both initial-condition and meteorological-forecast uncertainties peak, and steadily narrows for each successive initialization from December through June. This behavior suggests that water managers can interpret these bands as a realistic risk envelope, tightening operational thresholds as forecast lead times shorten.

Importantly, all these forecast gains were realized by applying the SES correction directly to the existing WRF + WEHY-HCM outputs, and no new physical-model components were introduced. This lightweight statistical post-processing layer adjusts persistent biases, requires only a modest historical training record, and can be added on top of the operational hydro-meteorological workflows without extensive retraining or tuning. Although this study does not include a direct comparison with other operational forecast products, a companion analysis^26^ showed that this forecasting methodology performs comparably to, and in some cases more accurately than, the current monthly flow forecasts issued by the California Department of Water Resources (DWR) through their Bulletin 120 forecasts. Furthermore, although advanced machine learning techniques such as LSTM have shown skill in regional post-processing applications^23^ the SES approach offers advantages in operational simplicity and interpretability owing to better transparency in the forecast error correction mechanism (Eq. 1). Such transparency could allow users to better understand how forecast errors are adjusted, an aspect that may be especially valuable in real-time water management contexts where clarity in model behavior is important.

However, several limitations should be considered when interpreting these results. First, our error-training period (2018–2023) spans only six water years, which may limit the SES filter’s ability to learn rare extreme-event patterns. Moreover, the model has been tested on only a single out-of-sample year (2024), which constrains our ability to draw firm conclusions about the robustness and long-term reliability of the forecasting system. Second, the structural errors in WRF downscaling, such as misrepresentation of orographic precipitation, can propagate into the hydrologic model and may not be fully corrected by SES. Third, we have not tested alternative statistical or machine-learning–based correction methods, so it remains unclear whether seasonal exponential smoothing is truly optimal for this basin.

Despite their wider confidence bands, early-season forecasts (November and December initializations) still offer valuable advance guidance by quantifying the range of possible inflows months ahead. Reservoir operators can use these preliminary estimates to set initial storage targets and develop contingency release plans, flood‐control agencies can begin preparing for high‐flow scenarios, and agricultural planners can form early expectations of seasonal water availability. As the season advances, from November through each successive initialization, uncertainty steadily narrows, allowing all these stakeholders to progressively refine reservoir operations, flood‐risk management, and irrigation schedules with greater precision. In this way, the SES‐corrected system delivers actionable forecasts at every stage of the water year.

For future work, extending the analysis to multiple water years will help assess the SES filter’s robustness under more diverse climatic conditions. Testing other bias-correction methods and integrating real-time snow-water‐equivalent (SWE) observations could further improve reliability. Finally, future work could augment the existing CFSv2 ensemble forecasts, used in this study, by incorporating additional seasonal‐forecast systems, e.g., ECMWF SEAS, and then applying the SES correction to each member. This multi‐model approach would broaden the range of forcing scenarios, potentially enhancing forecast accuracy and yielding more reliable, well‐characterized uncertainty intervals.

To conclude, the SES-corrected seasonal forecasting system provides a straightforward but effective improvement to the WRF–WEHY-HCM framework by reducing errors, minimizing bias, and producing well-aligned confidence intervals. While the results are specific to the UFRB and the modeling system used here, this approach shows promise for adaptation in other basins with similar forecasting setups.

## Methods

### Study area

**Fig. 4:**
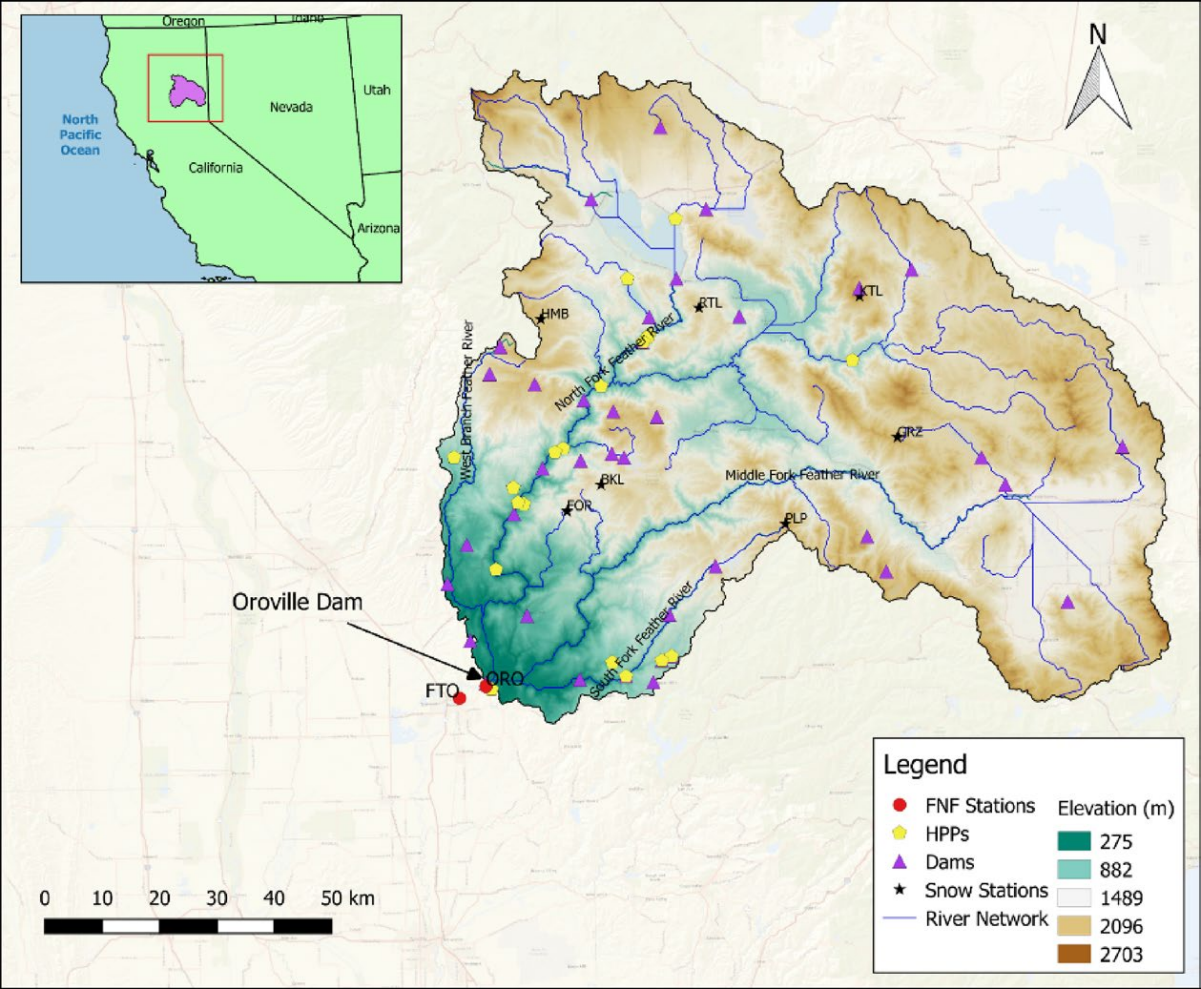
Digital elevation model (DEM) of the UFRB, overlaid with the locations of FNF stations, hydropower plants (HPPs), dams, and the river network. The map was generated using QGIS version 2.18.7^28^.

The UFRB covers approximately 9,334 square kilometers in the northern Sierra Nevada, where the range intersects the Cascade Range to the north and the Diamond Mountains to the east. Elevations in the UFRB range from 2,703 m near Lassen Peak to 275 m at Oroville Dam (Fig. 4). The region spans Plumas, Butte, Sierra, and Lassen counties, with most of the watershed lying within Plumas County. The population in the watershed is approximately 33,000 people^24^. It drains southwest to Lake Oroville, the largest reservoir in California’s State Water Project (SWP). Water from Lake Oroville supports irrigation, domestic use, and aquatic ecosystems in the Lower Feather River, Sacramento River, and Sacramento-San Joaquin Delta. The SWP provides water to over two-thirds of California’s population and delivers an average of 42.3 billion cubic meters annually for agriculture in the Central Valley^25^.

Oroville Dam, located at the southern edge of the watershed, is the tallest earth-fill dam in the United States, with a height of 235 m^27^. Lake Oroville, formed by the dam, has a storage capacity of 4.9 billion cubic meters and supplies 3.95 billion cubic meters annually as “firm” water to agricultural and urban users, primarily through export pumping from the Sacramento-San Joaquin Delta. The reservoir is fed by four main tributaries (Supplementary Table S4): the North Fork, Middle Fork, South Fork, and West Branch of the Feather River. The North Fork, the largest tributary, contains multiple dams and hydroelectric facilities, including PG&E’s 734 MW “Stairway of Power,” a system of ten hydroelectric powerhouses, eight dams, and an interconnected network of tunnels^25^.

The watershed has a Mediterranean climate with wet winters and dry summers. Precipitation varies significantly due to elevation, terrain, and aspect. The western slope of the watershed receives up to 2,300 millimeters of annual precipitation, while the Sierra Valley in the eastern portion receives as little as 280 millimeters. Snowpack in the Cascade-Sierra zone generates the highest runoff and groundwater recharge^25^.

### Atmospheric and hydrologic models used in the study

This study employs a coupled hydroclimate model known as the Watershed Environmental Hydrology Hydro-Climate Model (WEHY-HCM)^29^ to forecast seasonal inflows. The WEHY-HCM consists of two main components: an atmospheric model and a watershed hydrology model. The following subsections provide a brief description of the models used for each of these components.

#### Advanced weather research and forecasting model (WRF)

This study uses the Advanced Research Weather Research and Forecasting Model (WRF, version 3.9.1) for seasonal climate forecasting. The WRF model was developed through collaboration between the National Center for Atmospheric Research (NCAR), the National Oceanic and Atmospheric Administration (NOAA), and the Air Force Weather Agency (AFWA). It is a next-generation mesoscale numerical weather prediction system designed to meet both atmospheric research and operational forecasting needs. WRF is a non-hydrostatic, fully compressible model with a terrain-following vertical coordinate system, designed for both atmospheric research and forecasting^30^. It dynamically downscales coarse-resolution global atmospheric data, such as CFSv2 (∼100 km resolution), into fine-scale outputs. The WRF model produces outputs such as precipitation, temperature, and wind fields.

#### Watershed environmental hydrology (WEHY) model

The WEHY Model is a physically based model that uses the conservation of mass, momentum, and energy equations for water flow in both surface and subsurface flow domains of a watershed^31,32^. It includes a snow module and a hydrology module. The snow module computes snow surface temperature, depth, and melt by solving depth-averaged energy, mass, and density conservation equations. The *snow module* uses inputs like precipitation, air temperature, and radiation, which are typical outputs from Regional Climate Models (RCMs). It incorporates snow physics and solar geometry to estimate energy parameters, deriving solar angles from topographic maps created with DEM and GIS tools. This allows the module to simulate snowmelt processes while accounting for topography. The model outputs, such as daily SWE and snow depth distributions, are provided at a 100-meter spatial resolution. The *hydrologic module* divides the watershed into MCUs, primarily hillslopes, and integrates snow model outputs to simulate runoff and other hydrologic processes. This physically based module relies on geophysical, vegetation, and geomorphological data derived from GIS-based land and soil databases. The module simulates hydrologic processes by computing five components in parallel: unsaturated flow, subsurface stormflow, overland flow, groundwater flow, and channel flow. These computations provide flow discharge to the stream network and the underlying unconfined groundwater aquifer, which dynamically interacts with surface and subsurface hillslope processes. The input datasets used in constructing WEHY for the UFRB are given in Supplementary Table S5.

### Reconstruction of climatic and hydrologic conditions

To use the WEHY-HCM model for seasonal forecasting in the UFRB, the snow and hydrology modules must first be calibrated, requiring historical atmospheric data. Additionally, atmospheric forecasts are essential for predicting snow and inflow. In this study, Climate Forecast System Reanalysis (CFSR)^33^ data and NOAA’s CFSv2 9-month operational forecasts^34^ are used for historical data and atmospheric predictions, respectively. These datasets have coarse horizontal resolutions of approximately 40 km (CFSR) and 100 km (CFSv2) with 6-hour time intervals, which are unsuitable for watershed-scale hydrologic forecasting. Therefore, both datasets are dynamically downscaled to a finer resolution of 9 km with the WRF model in this study.

Three nested domains were selected for the WRF model to produce dynamically downscaled, high-resolution precipitation and associated atmospheric fields over the Sacramento River Basin. The horizontal resolutions of the domains were set to 81 km (Domain 1), 27 km (Domain 2), and 9 km (Domain 3). The WRF physics options applied in this study were based on prior studies conducted in the Sacramento River Basin^35,36^ which provide detailed descriptions of WRF calibration and validation, and physics options used for modeling.

Following the configuration and evaluation of the WRF model, WRF-reconstructed historical atmospheric data, derived from CFSR reanalysis, were used as inputs to the WEHY-HCM snow module. These inputs included variables such as temperature, relative humidity, wind speed, precipitation, and long- and short-wave radiation. In addition to atmospheric inputs, land condition data were also required for estimating snow accumulation and melt processes in the UFRB. Finally, the outputs from the snow model were integrated into the hydrology module of WEHY-HCM to reconstruct historical runoff and flow discharge for the UFRB.

### Calibration and validation of WEHY model for the UFRB

The snow and hydrology modules of the WEHY-HCM model must be calibrated before it can be used for seasonal forecasting in the UFRB. After obtaining the historical atmospheric data, these modules were calibrated and validated. This section details the calibration and validation processes for the WEHY model in the UFRB. Since the model comprises two components, the snow module and the hydrology module, their calibration and validation are discussed separately in two subsections. The calibration period spans the water years 2006 through 2016, while the validation period covers the water years 2017 through 2022.

#### Snow module

The model parameters and configurations of the snow module are specified in a control file. The parameters specified in the control file can be adjusted to calibrate the snow module of the WEHY model. In this study, adjustments were made to the following parameters: the wind speed correction factor, the bulk transfer coefficients for convective and latent heat transfer, the time constant for albedo, the converged albedo value, and the maximum albedo. These parameters were manually fine-tuned to ensure that the simulated SWE closely matched the observations from snow monitoring stations. Detailed explanation of these parameters is provided in Horne and Kavvas^37^. These parameters were modified until the simulated SWE aligned satisfactorily with the observed SWE at snow monitoring stations.

In this study, SWE data from seven snow stations were used to calibrate the snow model. The locations of these stations are shown in Fig. 4. The station name, station ID, county, and elevation information of these stations are given in Supplementary Table S6. All observational data were obtained directly from the California Data Exchange Center (CDEC) on a daily scale. The performance of the snow model was evaluated by comparing simulated point-scale SWE against daily observations for the period 2006–2016 at each of the seven stations. Once satisfactory model performance was achieved, the model was validated using data from 2017 to 2022. NSE values for snow were calculated only for time steps with available observation data. SI Fig. 7 and SI Fig. 8 illustrate the comparison between simulated snow model outputs and daily CDEC data for the calibration and validation periods, respectively.

The results show that higher elevation stations, located at elevations above 2000 m, generally show better model performance with high NSE values in the calibration period, e.g., KTL: NSE = 0.83, GRZ: NSE = 0.80. Higher elevations typically exhibit more stable snow accumulation due to colder temperatures, resulting in less frequent melting events. This likely enhances model accuracy. At lower elevations (e.g., FOR, BKL), warmer conditions and more complex snow-rain transitions reduce the model’s ability to replicate observed SWE accurately. At all snow monitoring stations, both during calibration and validation periods, the NSE and R^2^ values exceed 0.5, indicating satisfactory model performance.

#### Hydrology module

The WEHY model’s hydrology module relies on outputs from the snow module as inputs. Calibration of the module was performed manually and guided by monthly flow data. Calibration of this module involves adjusting parameters located in two distinct files: one for the hillslope model and another for the channel and meadow model. The hillslope runoff file allows for modifications to the global runoff characteristics of the watershed, while the channel and meadow file focuses on reach-specific parameters. In this study, over 100 simulations were conducted to assess the influence of these parameters on simulated flows by systematically adjusting them. Ultimately, six parameters in the hillslope runoff model file were altered, including summer and winter albedo, soil depth adjustment factor, summer and winter roughness length, and soil hydraulic conductivity adjustment factor. Additionally, three parameters in the channel and meadow model file were modified: the length of influence of the channel in the unconfined aquifer, the global factor for Manning’s roughness coefficient, and the global factor for water leakage from the bottom of the meadow.

The results were evaluated at the watershed outlet, corresponding to the inflow of Oroville Dam. To assess model performance, we used daily full natural flow (FNF) data provided by the California Data Exchange Center (CDEC). For water years prior to 2024, FNF data were reported at the ORO station (Oroville Dam). On January 10, 2024, CDEC moved the daily FNF sensor (sensor number 8) to the FTO station (Feather River at Oroville) and discontinued the daily FNF reporting at ORO. The FTO station is located just downstream of ORO and reflects nearly identical inflow conditions. We also verified that the daily FNF values from both stations are identical for overlapping periods. Accordingly, model calibration and validation were based on ORO data, while water year 2024 evaluation used FTO data, as this is now the only available source of daily FNF data from CDEC. The locations of these two stations are shown in Fig. 4.

**Fig. 5:**
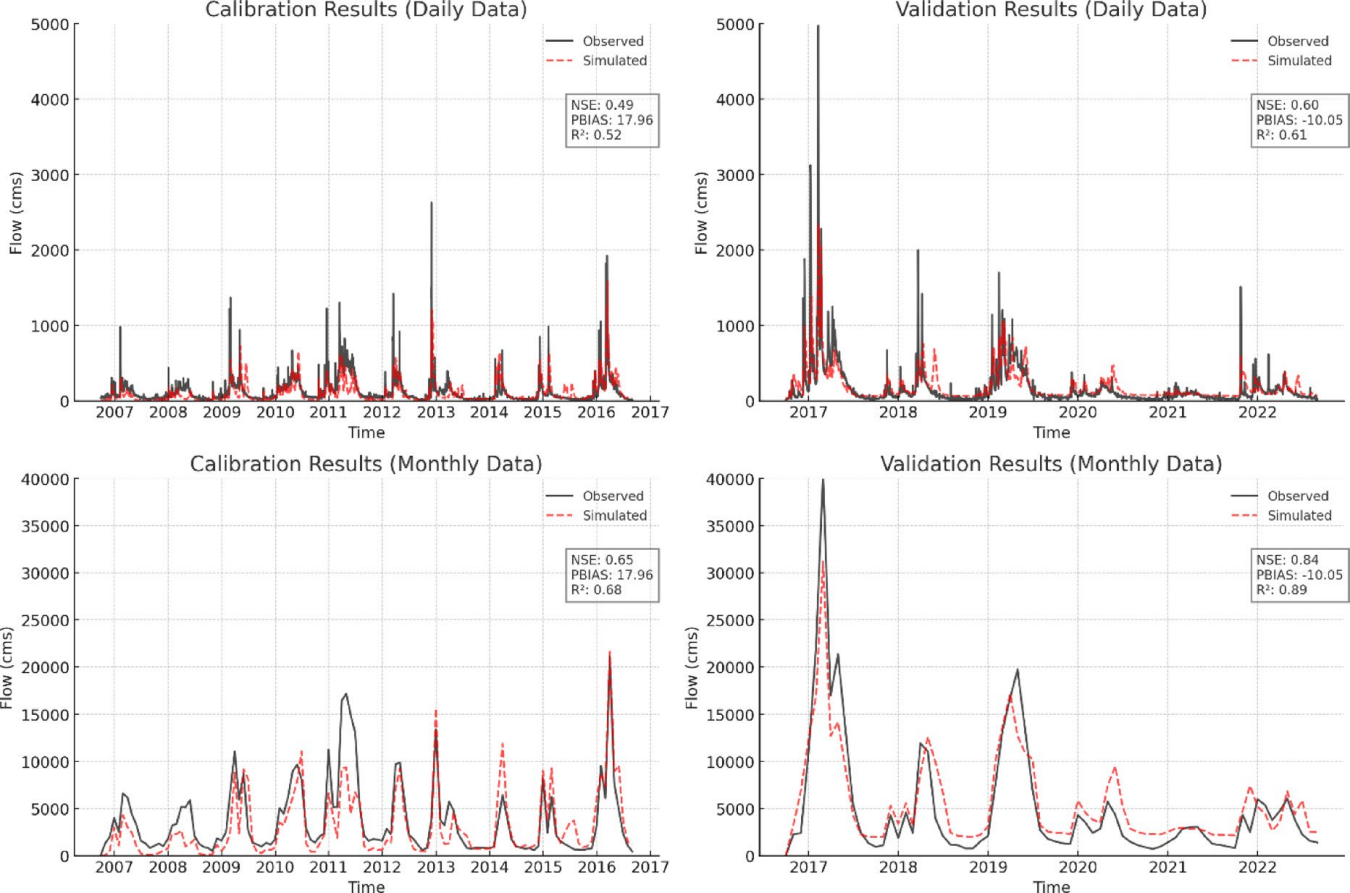
Calibration (left) and validation (right) of the WEHY model for Oroville Dam inflows, shown against daily (top) and monthly (bottom) Full Natural Flow (FNF) observations at the FTO^*^ station. Calibration period: 2007–2016; Validation period: 2017–2022.

The calibration and validation of the hydrology module were performed for the years 2007 through 2016 and 2017 through 2022, respectively, similar to the process used for the snow model. The results, shown in Fig. 5, indicate that during the calibration period, the NSE, PBIAS, and R² values calculated at the daily scale were slightly outside the generally accepted ranges for satisfactory model performance^38^ (i.e., NSE > 0.5, PBIAS ≤ ± 15%, and R² ≥ 0.70). On the other hand, these performance statistics refer to satisfactory model performance in the validation period, both at the daily and monthly scales. While the calibration period results showed some limitations, particularly in capturing peak flows, the model’s overall performance can still be considered satisfactory. This is especially true given that the focus of this study is on seasonal streamflow forecasting, rather than flood forecasting, where peak flow accuracy would be more critical. Moreover, the model’s performance indicators were only marginally outside the generally accepted thresholds for satisfactory performance during the calibration period. Therefore, the model’s performance is considered adequate for this study.

### Approach for seasonal streamflow forecasting

**Fig. 6:**
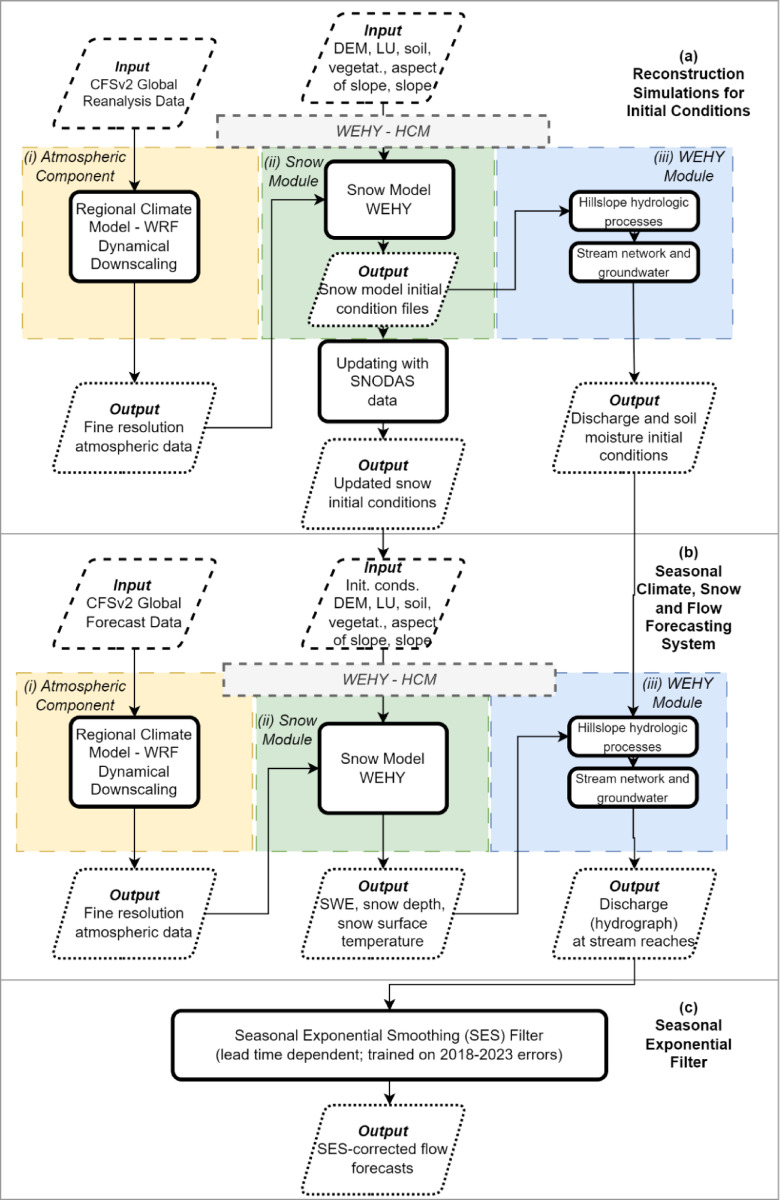
Hybrid seasonal streamflow forecasting workflow: (**a**) Initial-condition reconstruction via WRF downscaling of CFSv2 reanalysis, WEHY snow/hydrology modeling, and SNODAS updates; (**b**) Seasonal forecasting by WRF downscaling of global forecasts into WEHY snow and flow modules; (**c**) Lead‐time–dependent SES correction—trained on 2018–2023 hindcast errors—to produce bias‐adjusted inflow forecasts with quantified uncertainty.

The Seasonal Streamflow Forecasting System for the Sacramento River Basin developed by Kavvas et al. (2025)^26^ comprises three main components (Fig. 6): (a) Reconstruction simulations for initial conditions, (b) Deterministic forecasting of seasonal streamflow, and (c) Statistical updating of this streamflow using seasonal exponential smoothing. Part (a) has previously been described. The following subsections provide detailed explanations of parts (b) and (c) of the methodology.

#### Deterministic seasonal streamflow forecasting

The deterministic forecasting component of the seasonal streamflow forecasting system consists of three key elements (Fig. 6 – b): (i) a seasonal climate forecasting system, (ii) a seasonal snow forecasting system, and (iii) a seasonal streamflow forecasting system. The climate forecasting system uses the Weather Research and Forecasting (WRF) model^30^ to dynamically downscale operational forecasts from the Climate Forecasting System version 2 (CFSv2)^34^. The CFSv2 data, initially at a coarse resolution of ~ 100 km, are downscaled to a finer resolution of 9 km with hourly intervals, tailored specifically to the Sacramento River Basin. The snow and flow forecasting components are built on the Watershed Environmental Hydrology-Hydro-Climate Model (WEHY-HCM)^29^which integrates atmospheric and hydrologic processes at the watershed scale. The snow forecasting system employs WEHY-HCM’s snow model, driven by WRF-generated climate forecasts (listed in Supplementary Table S7), processed at an hourly time step with a 9 km resolution. The inflow forecasting system uses WEHY-HCM to transform snow model outputs into hydrographs for specified stream locations. These hydrographs, representing flow at the outlets of the Sacramento River subbasins, serve as the system’s final outputs. The following subsections briefly describe the three elements of the deterministic forecasting component.

##### Seasonal climate forecasting system

The WRF model generates an ensemble of seasonal climate forecasts by using CFSv2 data from fifteen consecutive days within a given month, specifically from the 16th to the 30th. Limiting the ensemble to the last fifteen members of the initialization month balances the need for up-to-date atmospheric information with the practical constraints of computation and data processing. If data for any day in this range is unavailable, it is substituted with data from the preceding day(s) to ensure a complete fifteen-day dataset. Each individual forecast, referred to as a forecast member, contributes to forming a fifteen-member ensemble. This ensemble provides seasonal climate forecasts from the following month through to the end of July of the water year. Ensemble forecasts are produced for each initialization month, from November through June. For instance, the ensemble initialized in January, derived from fifteen consecutive forecast members, offers predictions starting in February and extending through July. Similarly, ensembles initialized in February, March, April, May, and June follow the same six-month lead-time window. Consequently, because CFSv2 provides up to six months of forecasts, ensembles initialized in November and December extend through May and June, respectively. Monthly updates are made as fifteen new forecast members are added, ensuring forecasts remain current and account for evolving conditions.

##### Seasonal snow forecasting system

Using the WEHY-HCM model, snow forecasts are generated based on climate data from individual seasonal climate forecast members, forming a fifteen-member ensemble for each month from November to June. To address the need for snow initial conditions, simulations during calibration and validation typically start on October 1 st, the beginning of the water year, when no snow is present in the target basins. However, in the forecast system, hindcasts begin mid-season when snow is already present, requiring initial conditions to be generated separately.

Initial snow conditions are produced using two datasets: CFSR reanalysis data and SNODAS data^39^. For CFSR, the snow model simulates processes starting on October 1 st using downscaled atmospheric data generated by the WRF model. During this simulation, daily snow initial condition files are created, and the file corresponding to the starting date of the hindcast is selected for use. For SNODAS, snow depth data are extracted and interpolated onto the snow model grids. The interpolated snow depth data then replace the corresponding values in the CFSR-based initial condition file. These updated files serve as initial conditions for the snow model, which is run for each forecast member to produce an ensemble of seasonal snow forecasts.

##### Seasonal streamflow forecasting system

The WEHY model uses snow forecast results to generate streamflow forecasts, requiring initial conditions similar to the snow model. To establish these conditions, the WEHY hillslope and river channel routing models are run with inputs from the earlier snow reconstruction simulations to determine soil moisture and inflow data at the forecast start date. These initial conditions are then used to run the WEHY model for each forecast member, producing an ensemble of seasonal streamflow forecasts for each basin.

#### Statistical updating of seasonal streamflow using seasonal exponential smoothing

The numerical atmospheric-hydrologic modeling system used for deterministic forecasting in the Sacramento River Basin provides valuable insights into seasonal streamflow. However, as demonstrated in its application to the UFRB (see Section 3.1), the monthly forecasts produced by this system can deviate significantly from observed values. These discrepancies primarily arise from the uncertainty in the ensemble forecasts of the global CFSv2, which operates at a coarse resolution. This uncertainty is compounded by the nonlinear dynamics of global climate conditions over the six-month forecast period. To address these limitations, a lead time-dependent seasonal exponential smoothing (SES) filter was developed to correct forecast errors and improve accuracy (Fig. 6 – c). By combining these corrected errors with the original forecasts, the system achieves enhanced seasonal streamflow forecasts.

We applied seasonal exponential smoothing to the series of streamflow hindcast errors, using 2018–2023 as our training period to fit the model for the UFRB. We reserved 2024 as a testing period to evaluate its out-of-sample forecasting performance. Once fitted, the model produced error forecasts for 2024, which we then applied to adjust the deterministic streamflow forecasts for that year.

A seasonal exponential-smoothing filter is applied to the monthly hindcast errors at each lead time (for l = 1, 2,…,6), defined by

1 $$\:{S}_{t|l}=\left\{\begin{array}{c}{\epsilon\:}_{t|l}\:\:\:\:\:\:\:\:\:\:\:\:\:\:\:\:\:\:\:\:\:\:\:\:\:\:\:\:\:\:\:\:\:\:\:\:\:\:\:\:\:\:\:\:\:\:\:\:\:\:\:\:\:\:\:\:\:\:\:\:\:\:\:\:\:\:t\le\:12,\:\\\:\alpha\:{\epsilon\:}_{t-12|l}+\left(1-\alpha\:\right){S}_{t-12|l}\:\:\:\:\:\:\:\:\:\:\:\:\:\:\:\:\:\:\:\:\:\:\:\:\:\:t>12,\end{array}\right.$$

and the resulting smoothed value $\:{S}_{t|l}$ serves as the -step-ahead error forecast $\:{\widehat{\epsilon\:}}_{t+12|l}$. The single smoothing parameter is calibrated by minimizing the sum of squared differences between the observed and smoothed error forecasts. Details of the methodology are provided in Kavvas et al. (2024).

## Supplementary Information

Below is the link to the electronic supplementary material.

Supplementary material 1 (PNG 289.2 kb)

Supplementary material 2 (PNG 296.3 kb)

Supplementary material 3 (PNG 254.7 kb)

Supplementary material 4 (PNG 221.6 kb)

Supplementary material 5 (PNG 250.9 kb)

Supplementary material 6 (PNG 311.8 kb)

Supplementary material 7 (PNG 585.2 kb)

Supplementary material 8 (PNG 562.0 kb)

Supplementary material 9 (PDF 2270.8 kb)

Supplementary material 10 (DOCX 24.9 kb)

## Data Availability

All data generated or analysed during this study are included in this published article [and its supplementary information files].
