# Supplementary figures and images for: Hybrid physical–statistical framework for seasonal streamflow forecasting in the Upper Feather River Basin, California
Source: Sci Rep. 2025 Aug 30;15:31968. doi: 10.1038/s41598-025-15932-7 (PMC12398547; doi:10.1038/s41598-025-15932-7)

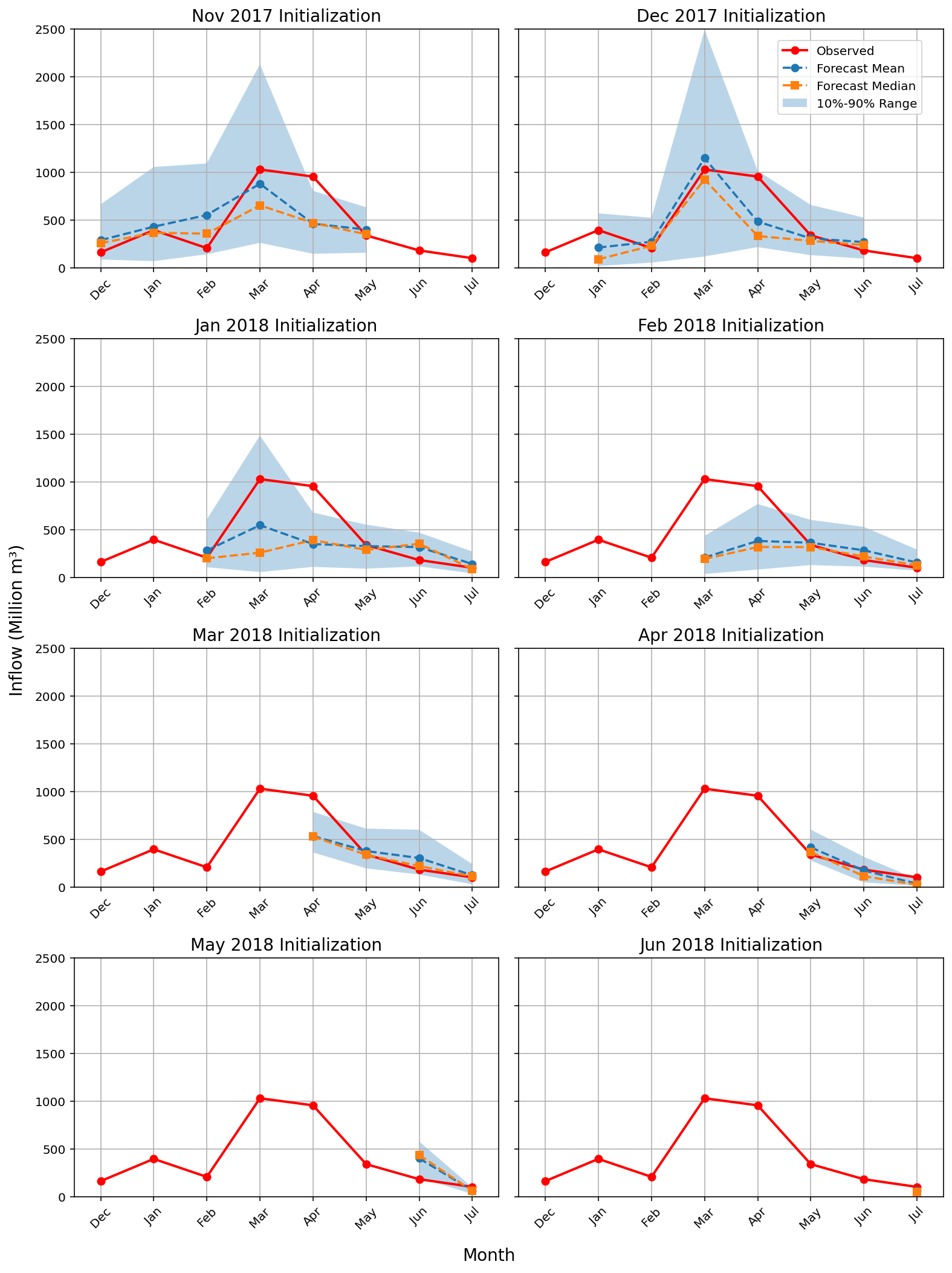

Supplement: Supplementary file 1 — Supplementary material 1 (PNG 289.2 kb) [file 41598_2025_15932_MOESM1_ESM.png]

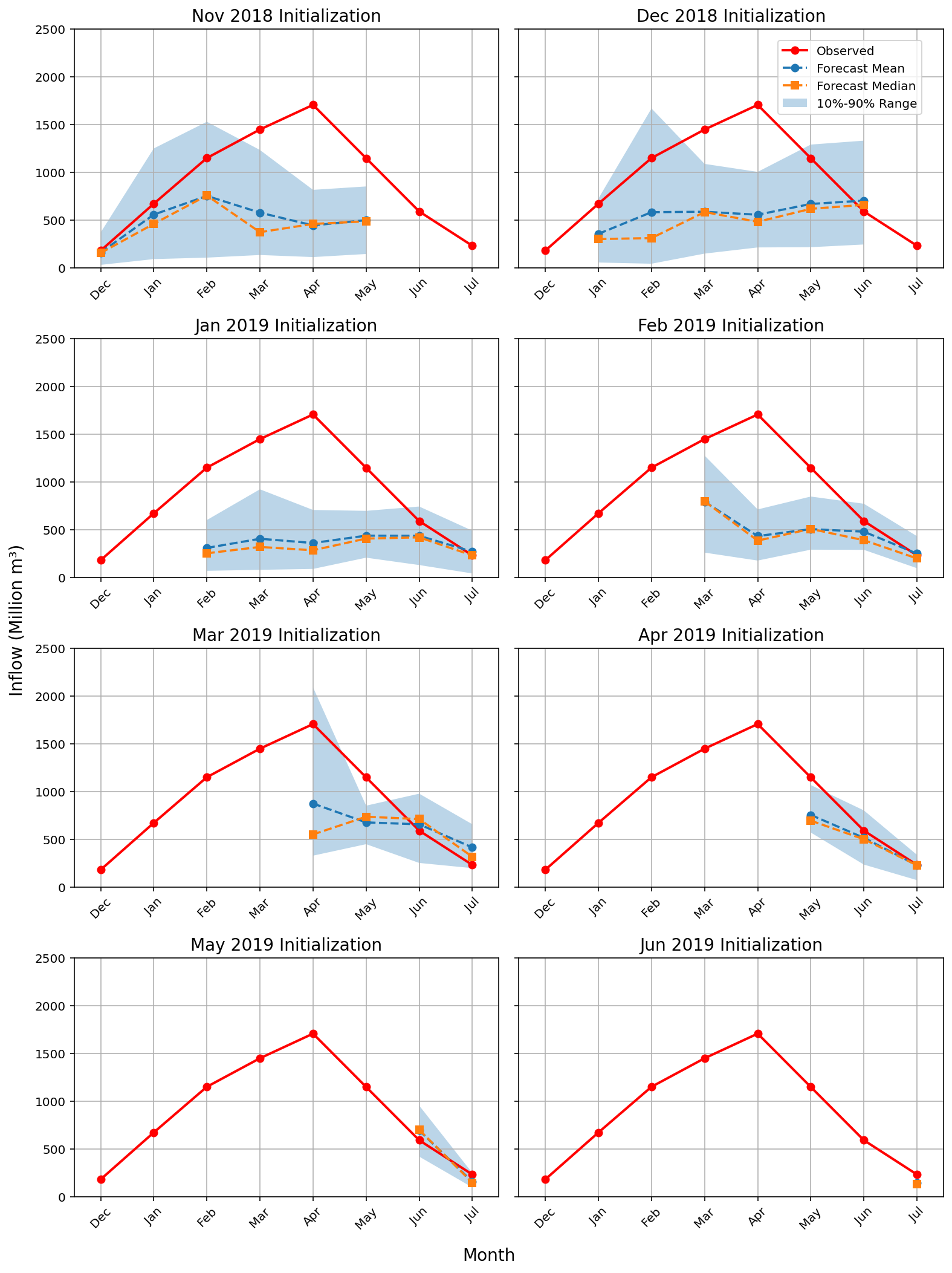

Supplement: Supplementary file 2 — Supplementary material 2 (PNG 296.3 kb) [file 41598_2025_15932_MOESM2_ESM.png]

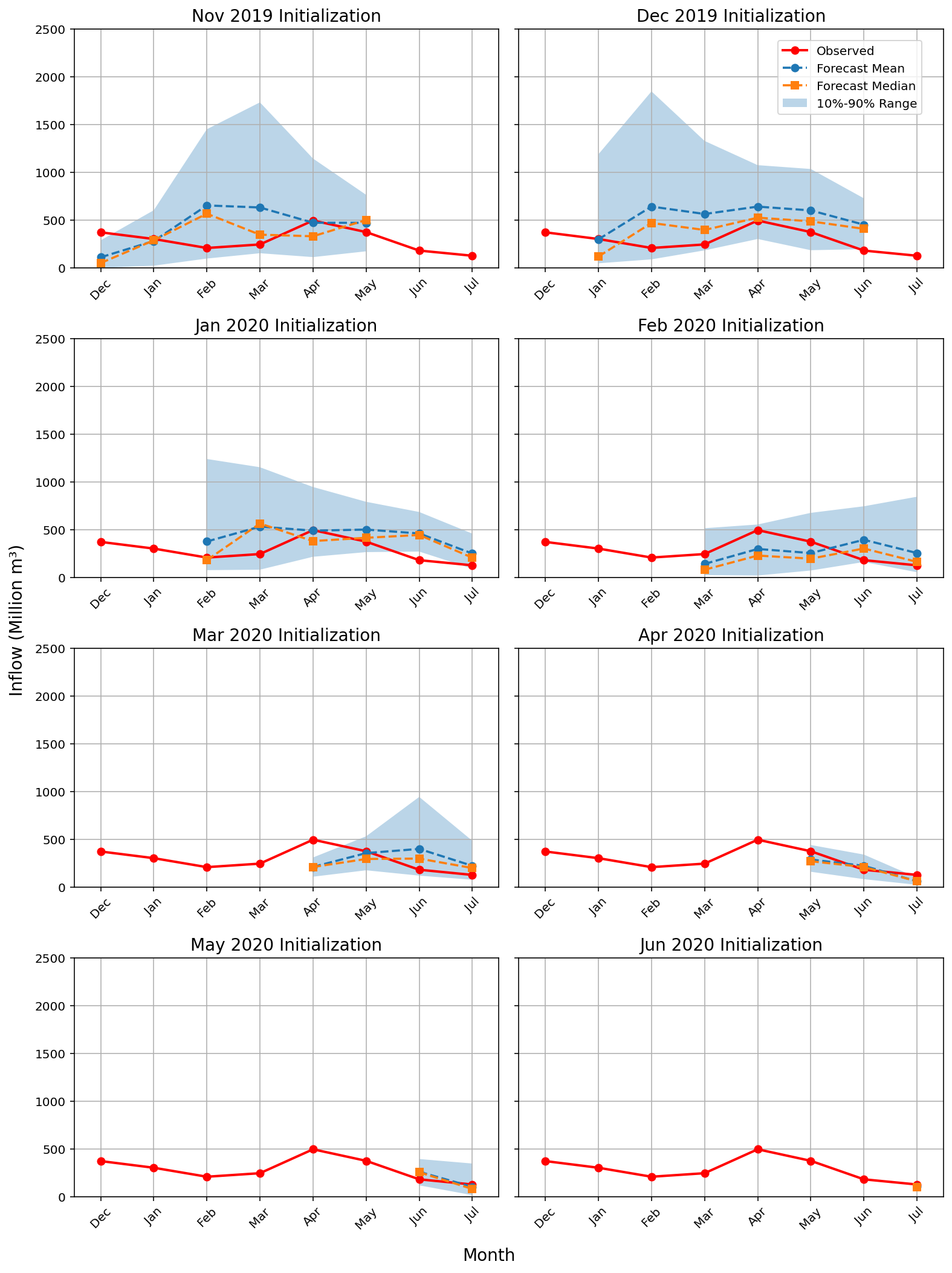

Supplement: Supplementary file 3 — Supplementary material 3 (PNG 254.7 kb) [file 41598_2025_15932_MOESM3_ESM.png]

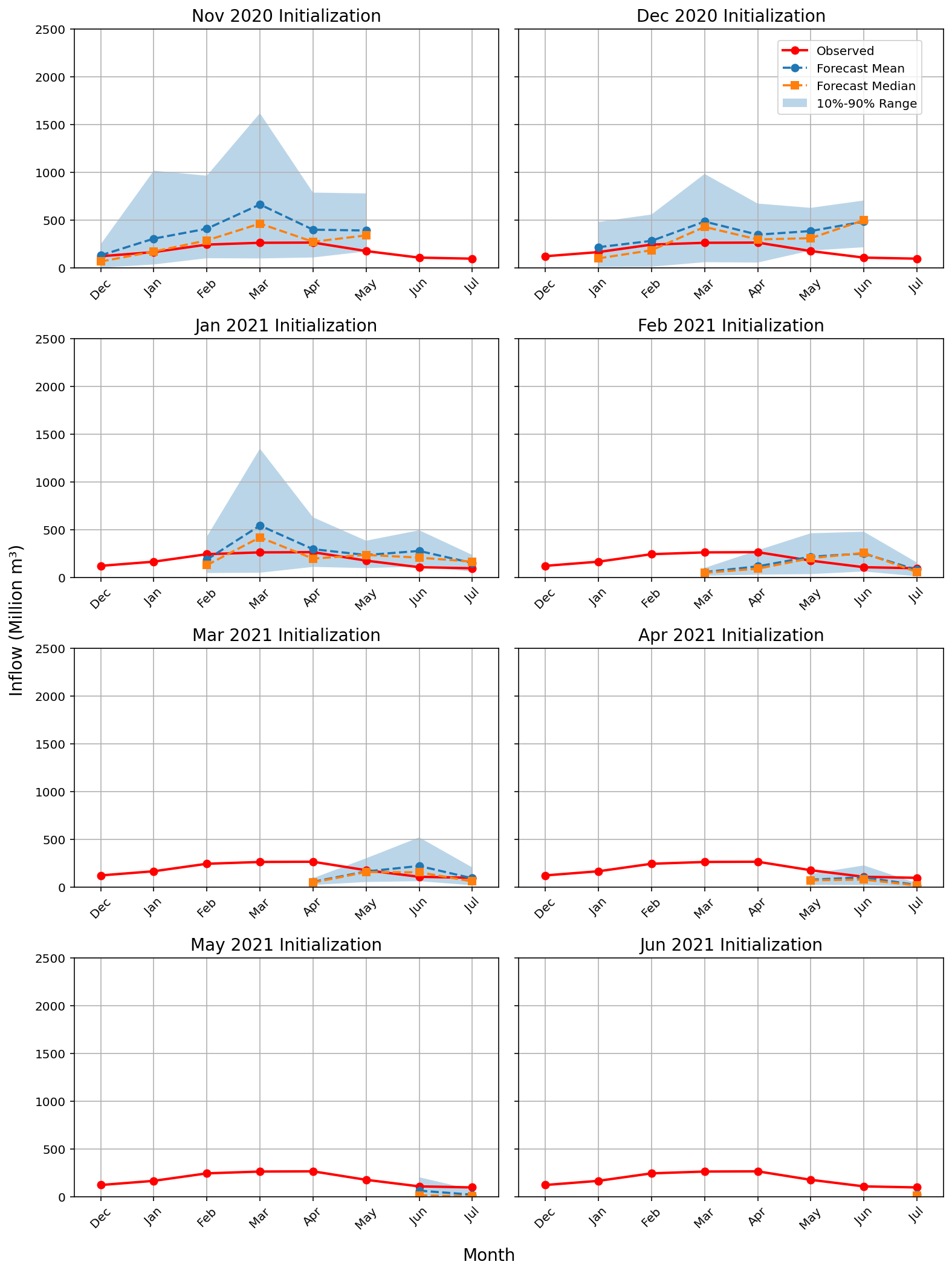

Supplement: Supplementary file 4 — Supplementary material 4 (PNG 221.6 kb) [file 41598_2025_15932_MOESM4_ESM.png]

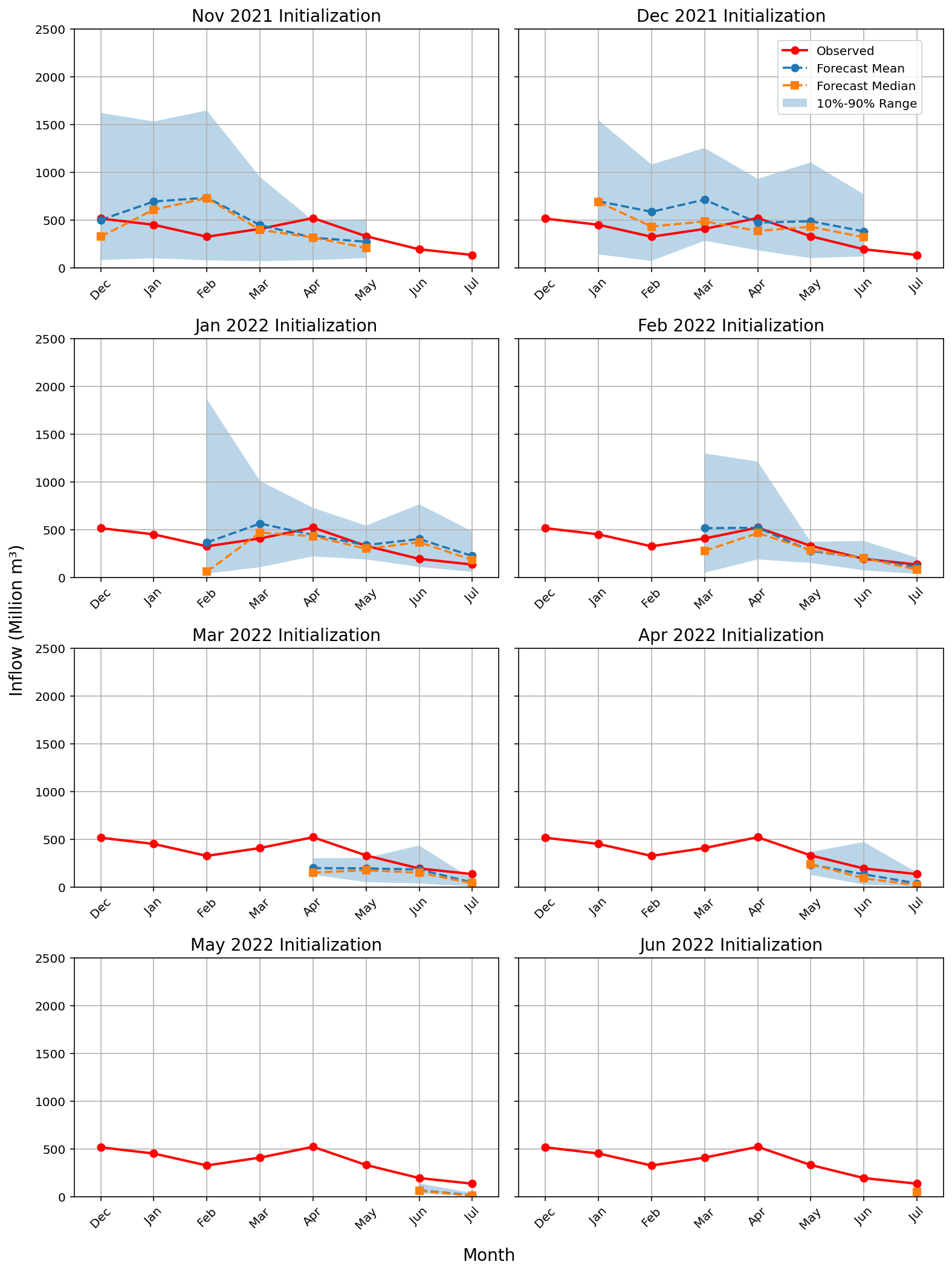

Supplement: Supplementary file 5 — Supplementary material 5 (PNG 250.9 kb) [file 41598_2025_15932_MOESM5_ESM.png]

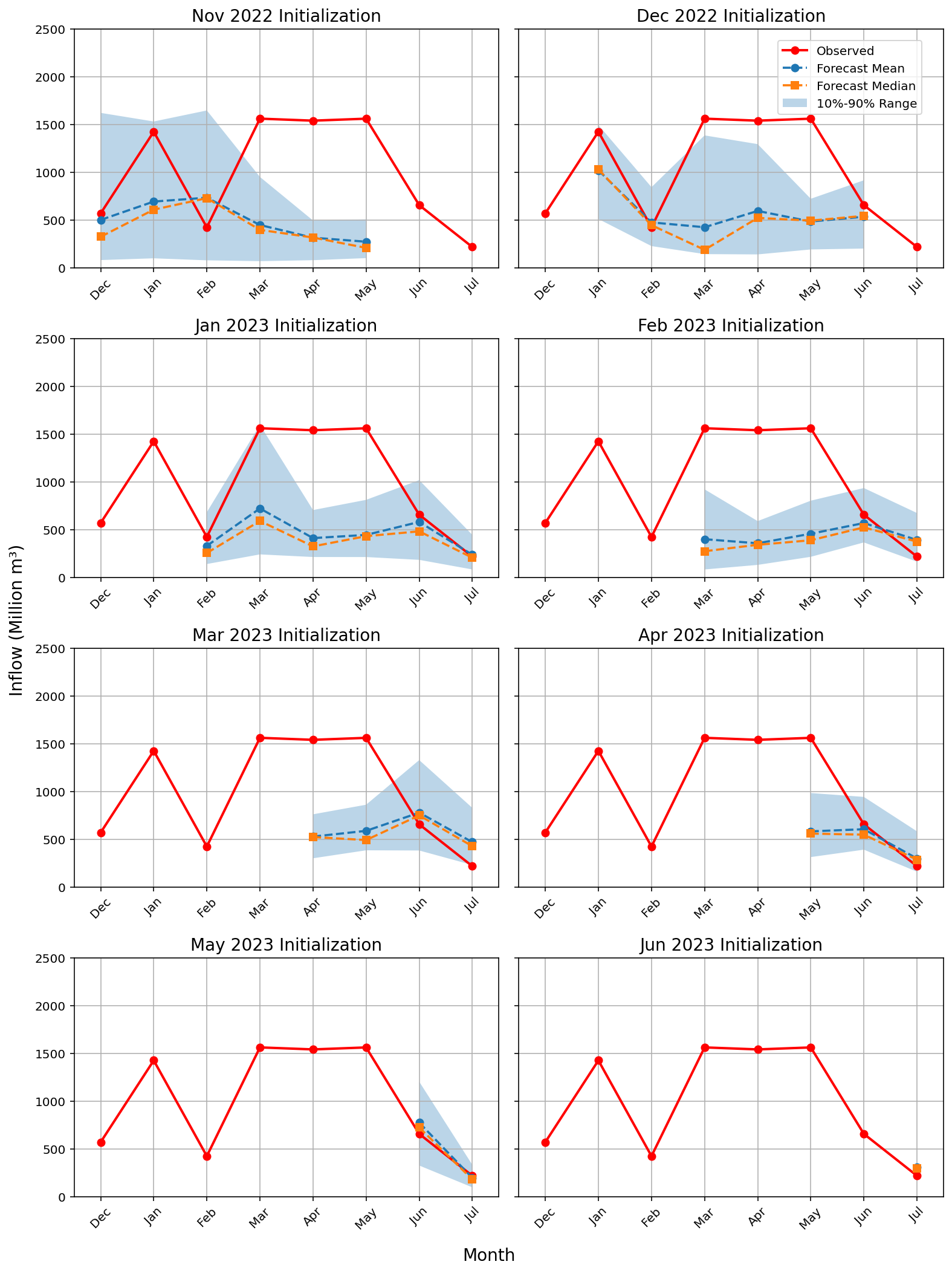

Supplement: Supplementary file 6 — Supplementary material 6 (PNG 311.8 kb) [file 41598_2025_15932_MOESM6_ESM.png]

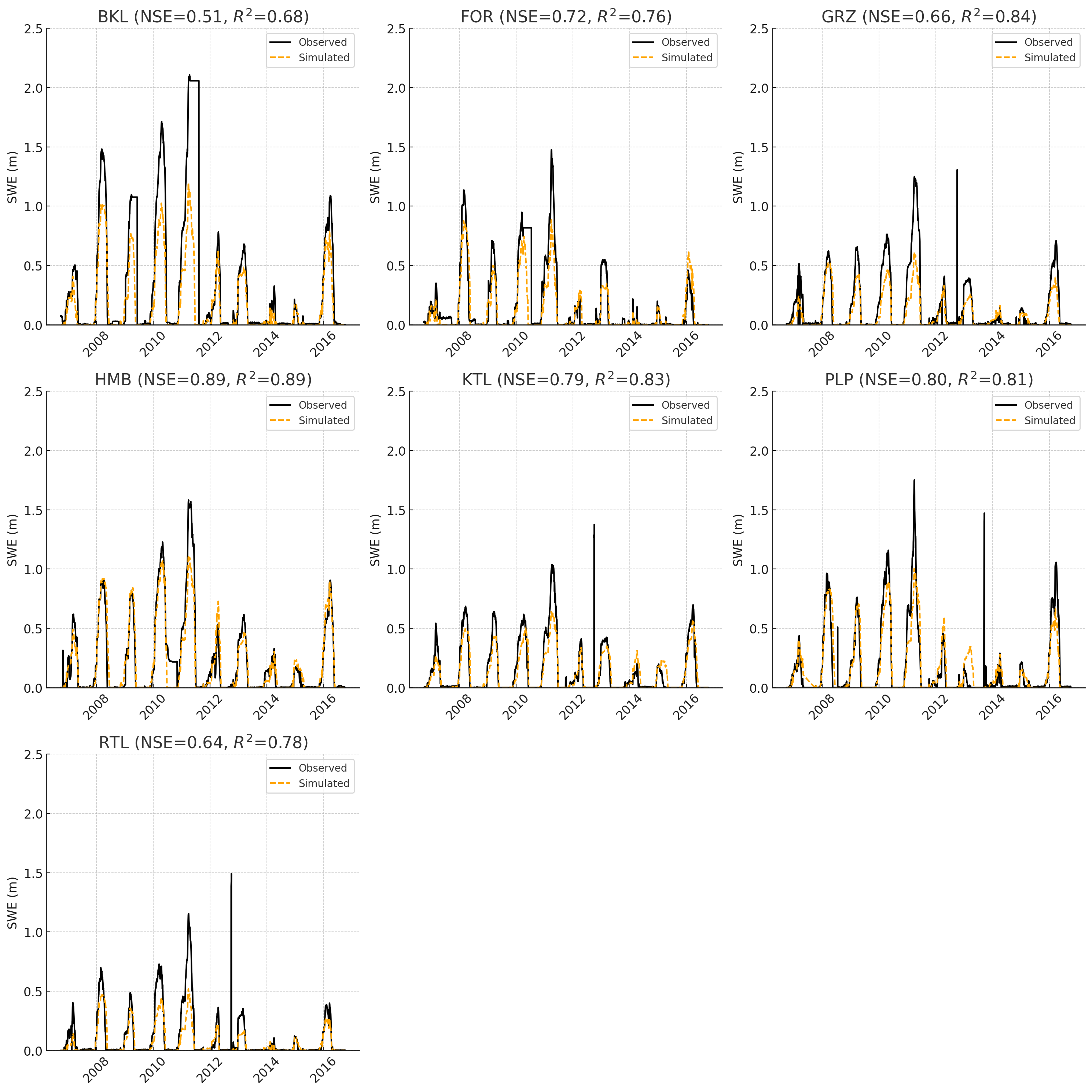

Supplement: Supplementary file 7 — Supplementary material 7 (PNG 585.2 kb) [file 41598_2025_15932_MOESM7_ESM.png]

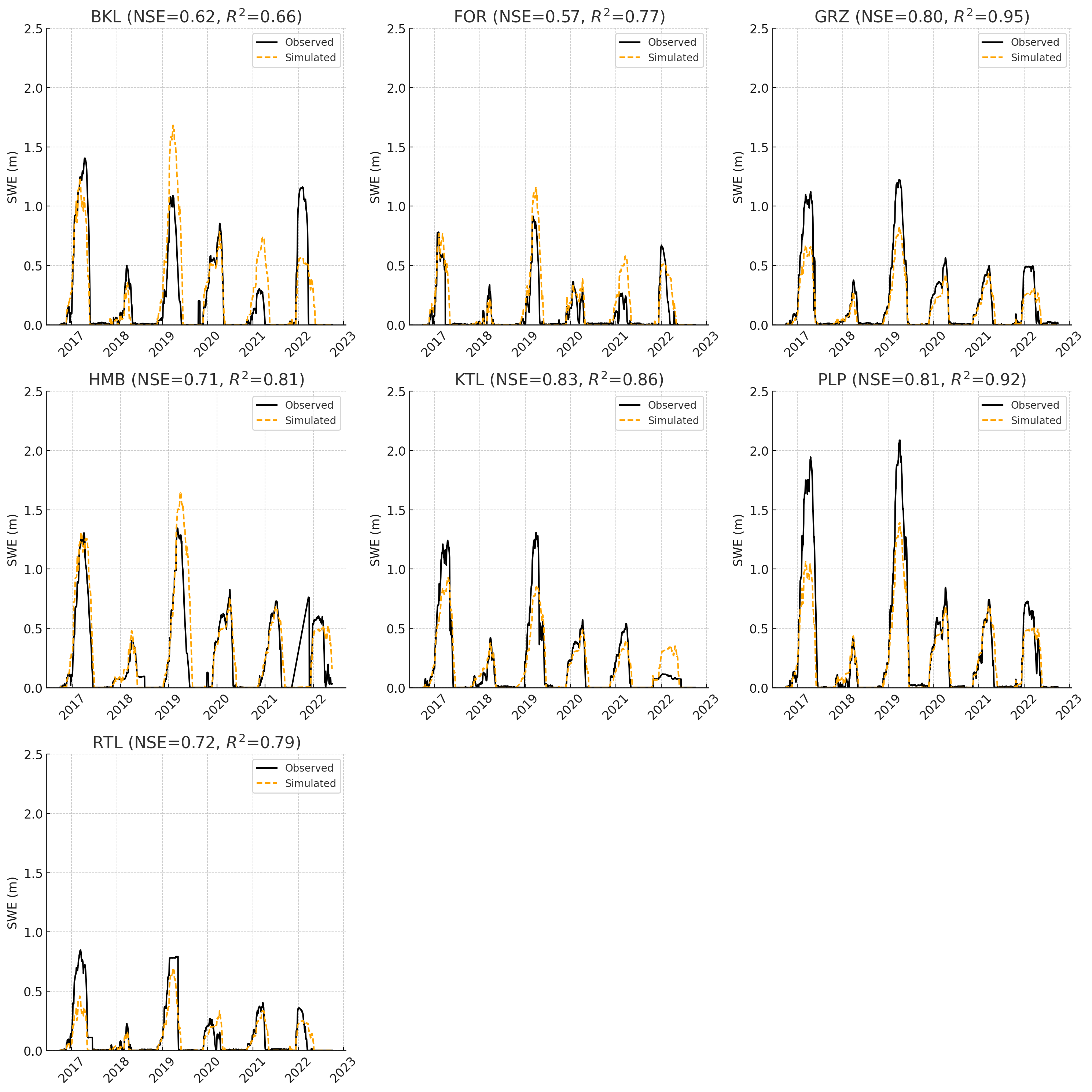

Supplement: Supplementary file 8 — Supplementary material 8 (PNG 562.0 kb) [file 41598_2025_15932_MOESM8_ESM.png]
