# Supplementary material for: Hybrid physical–statistical framework for seasonal streamflow forecasting in the Upper Feather River Basin, California
Source: Sci Rep. 2025 Aug 30;15:31968. doi: 10.1038/s41598-025-15932-7 (PMC12398547; doi:10.1038/s41598-025-15932-7)
Supplement: Supplementary file 9 — Supplementary material 9 (PDF 2270.8 kb) [file 41598_2025_15932_MOESM9_ESM.pdf]

Hybrid Physical–Statistical Framework for Seasonal Streamflow Forecasting in the Upper Feather River Basin, California

Z. Ozcan[1], Y. Iseri[1,2], F. Ulloa[1], N. Imbulana[1], E. Snider[1], M. Mure-Ravaud[1], M. L. Anderson[3], M. L. Kavvas[1]

[1]Hydrologic Research Laboratory, Department of Civil & Envr. Engineering, University of California, Davis, California, United States

[2]Now at the Arid Land Research Center, International Platform for Dryland Research and Education, Tottori University, Japan

[3]California Department of Water Resources, Sacramento, California, United States

Supplementary Information

Table S1 Initialization months and corresponding forecast lead times (in months) along with the forecasted calendar months.

**Initialization month**	**Lead Times**					
	**1**	**2**	**3**	**4**	**5**	**6**
	**Forecasted Months**					
**Nov**	Dec	Jan	Feb	Mar	Apr	May
**Dec**	Jan	Feb	Mar	Apr	May	Jun
**Jan**	Feb	Mar	Apr	May	Jun	Jul
**Feb**	Mar	Apr	May	Jun	Jul	
**Mar**	Apr	May	Jun	Jul		
**Apr**	May	Jun	Jul			
**May**	Jun	Jul				
**Jun**	Jul					

Table S2 RMSE (million m^3) and PBIAS (%) for each forecast initialization month for the deterministic forecasting system.

	**Nov-23 init.**	**Dec-23 init.**	**Jan-24 init.**	**Feb-24 init.**	**Mar-24 init.**	**Apr-24 init.**	**May-24 init.**	**Jun-24 init.**
**RMSE**	332.3	410.3	323.9	417.5	378.6	338.7	160.1	156.8
**PBIAS**	12.6	42.1	33.0	34.4	26.9	49.6	66.2	90.6

Table S3 RMSE (million m^3) and PBIAS (%) for each forecast initialization month for the SES-corrected forecasts.

	Nov-23 init.	Dec-23 init.	Jan-24 init.	Feb-24 init.	Mar-24 init.	Apr-24 init.	May-24 init.	Jun-24 init.
**RMSE**	271.0	156.6	143.4	99.2	151.9	186.7	151.4	135.0
**PBIAS**	-10.0	8.0	8.6	-5.0	24.5	30.7	58.0	78.0

Table S4 Key characteristics of the four main tributaries of Lake Oroville.

Major Division	Area (km²)	Percent of Watershed Area	Mean Daily Flow (m³/s)*	Mean Annual Inflow (hm³)	Percent of Annual Total
West Branch	429	4.6	9.9	312.6	6.6
North Fork	5582	59.8	91.5	2884.4	60.4
Middle Fork	2996	32.1	42.5	1339.5	28.1
South Fork	327	3.5	7.4	232.2	4.9
Total	9334	100	151.2	4768.7	100

*Source: California Department of Water Resources

Table S5 Input datasets used by the WEHY model for the UFRB.

Data Type	Source	Spatial Resolution
Digital Elevation Model (DEM)	U.S. Geological Survey (USGS)	1 arc-second
Land Use / Land Cover	FRAP 2006 Statewide Land Use / Land Cover Mosaic (formerly CaSIL 2010)	30 m
Soil	USDA NRCS “Web Soil Survey” (STATSGO2, 2013)	Varies by map unit
Leaf Area Index (LAI)	MODIS MOD15A2H (Terra/Aqua, average)	1 km

Table S6 Characteristics of snow survey stations used for calibrating the snow model.

Station Name	STA ID	County	Elevation (m)
Bucks Lake	BKL	Plumas	1790
Four Tress	FOR	Plumas	1586
Grizzly Ridge	GRZ	Plumas	2103
Humbug	HMB	Plumas	1981
Kettle Rock	KTL	Plumas	2225
Pilot Peak (DWR)	PLP	Plumas	2073
Rattlesnake	RTL	Plumas	1893

Source: California Data Exchange Center (CDEC)

Table S7 WRF output variables used as input to the snow model.

**WRF output variable**	**Units**
Precipitation	mm
Surface pressure – top pressure	Mb = hPa
Potential temperature	K
Mixing ratio at the first layer in WRF	g/kg
Wind speed at the first layer in WRF	m/s
Geopotential height	m
Downward shortwave radiation	W/m^2
Downward longwave radiation	W/m^2
Air temperature at the first layer in WRF	K
u component of wind velocity at 10 m	m/s
v component if wind velocity at 10 m	m/s
Mixing ratio at 2 m	g/kg
Air temperature at 2 m	^0C

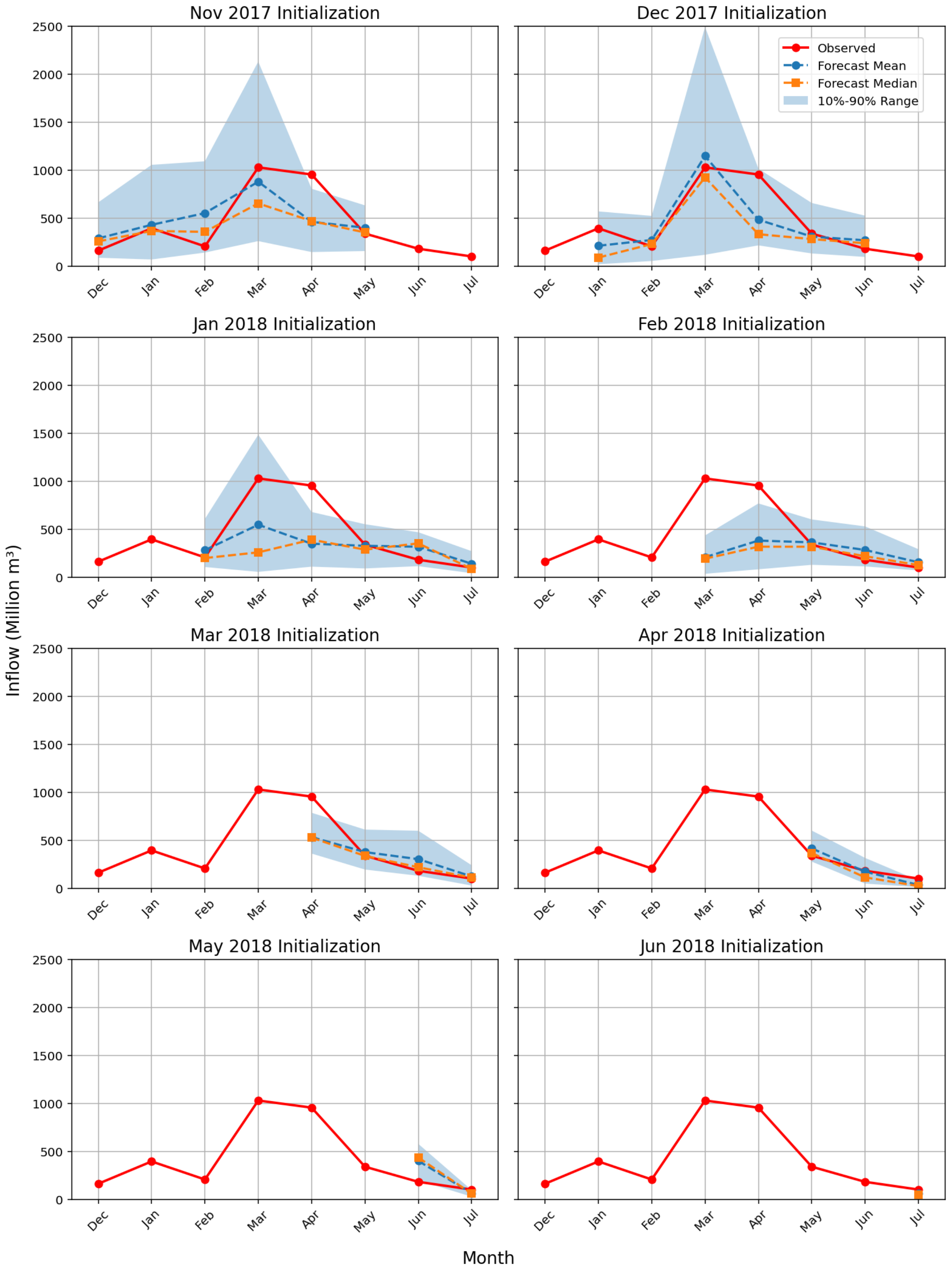

Fig. S 1 Deterministic seasonal streamflow forecasts to the FTO station for water year 2018 in the UFRB. Each subplot corresponds to a different forecast initialization month (Nov–Dec 2017; Jan–Jun 2018) and displays observed inflows from Dec 2017 through Jul 2018 (red circles), forecast mean (blue circles), forecast median (orange squares), and the 10 %–90 % exceedance range (shaded band). The common x-axis spans Dec through Jul, and the y-axis shows inflow in million m³.

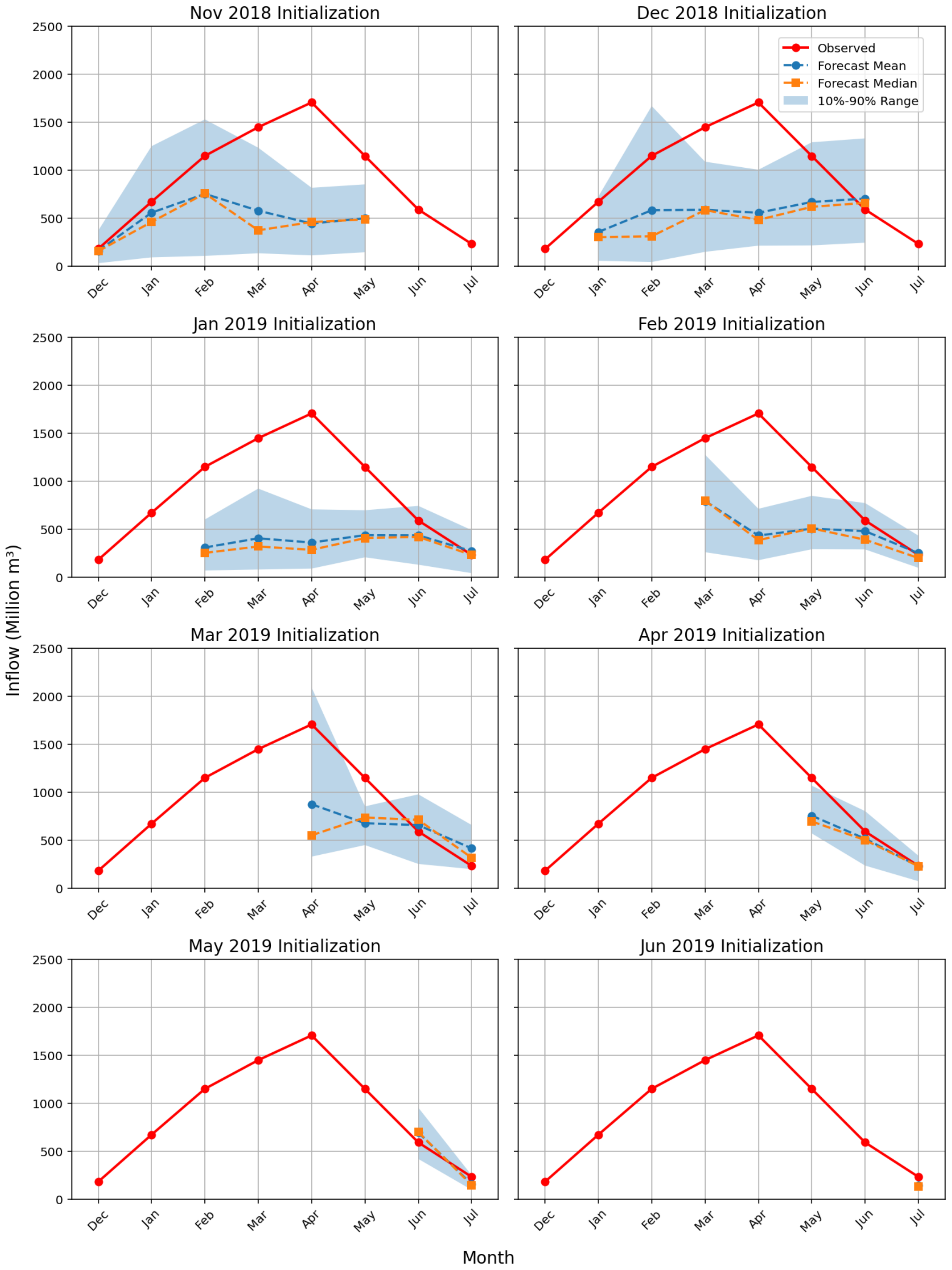

Fig. S 2 Deterministic seasonal streamflow forecasts to the FTO station for water year 2019 in the UFRB. Each subplot corresponds to a different forecast initialization month (Nov–Dec 2018; Jan–Jun 2019) and displays observed inflows from Dec 2018 through Jul 2019 (red circles), forecast mean (blue circles), forecast median (orange squares), and the 10 %–90 % exceedance range (shaded band). The common x-axis spans Dec through July, and the y-axis shows inflow in million m³.

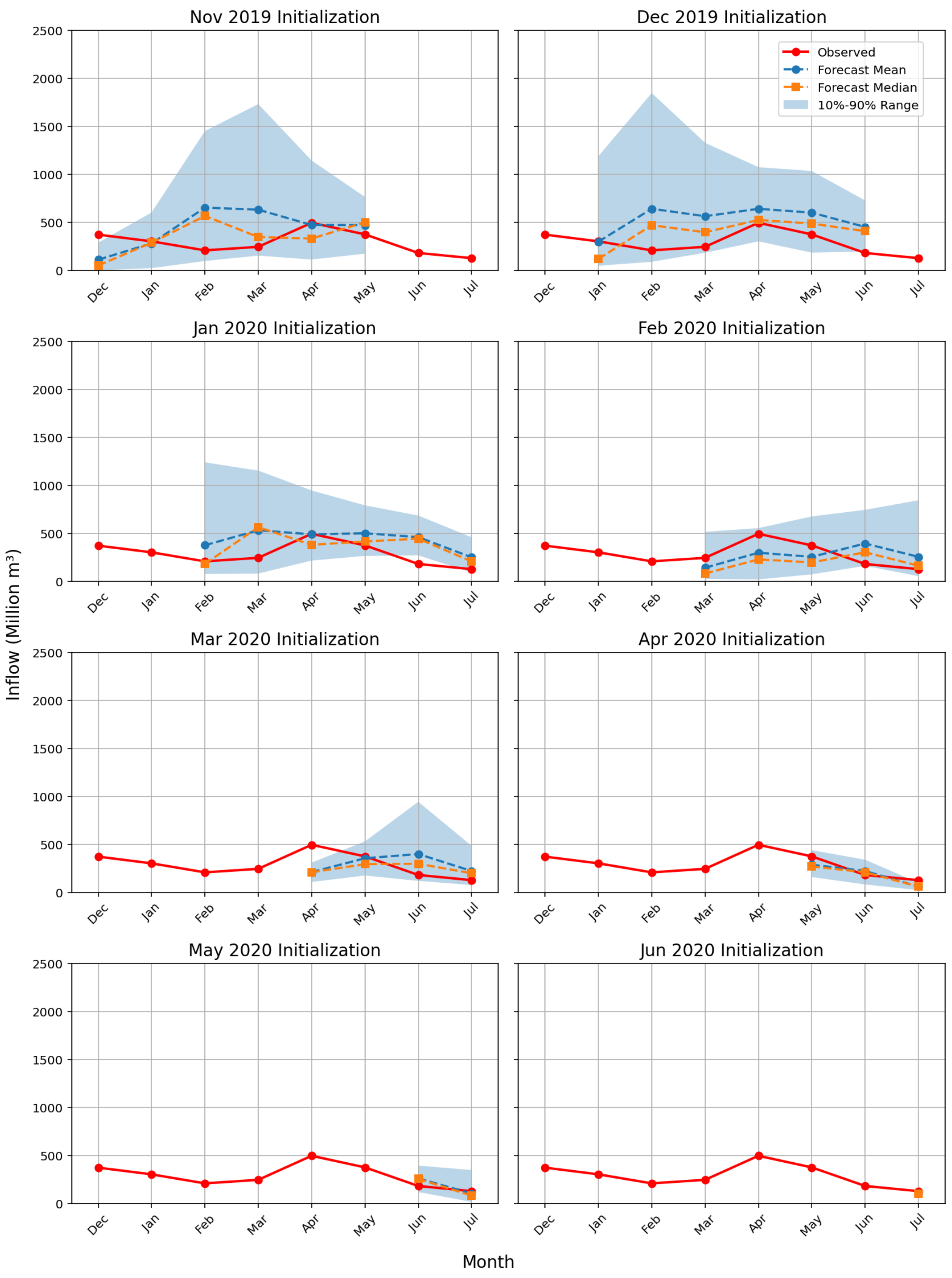

Fig. S 3 Deterministic seasonal streamflow forecasts to the FTO station for water year 2020 in the UFRB. Each subplot corresponds to a different forecast initialization month (Nov–Dec 2019; Jan–Jun 2020) and displays observed inflows from Dec 2019 through Jul 2020 (red circles), forecast mean (blue circles), forecast median (orange squares), and the 10 %–90 % exceedance range (shaded band). The common x-axis spans Dec through Jul, and the y-axis shows inflow in million m³.

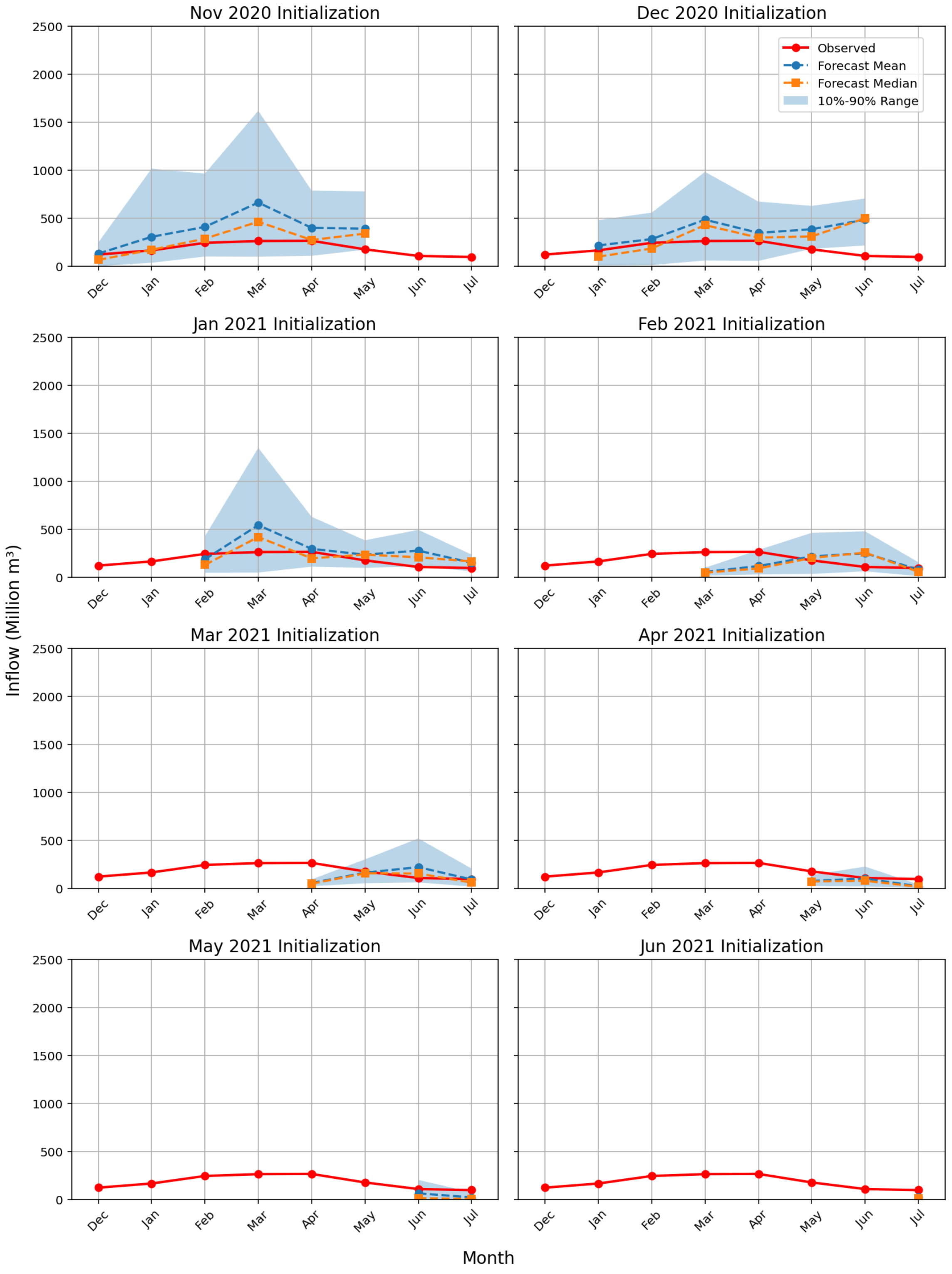

Fig. S 4 Deterministic seasonal streamflow forecasts to the FTO station for water year 2021 in the UFRB. Each subplot corresponds to a different forecast initialization month (Nov–Dec 2020; Jan–Jun 2021) and displays observed inflows from Dec 2020 through Jul 2021 (red circles), forecast mean (blue circles), forecast median (orange squares), and the 10 %–90 % exceedance range (shaded band). The common x-axis spans Dec through Jul, and the y-axis shows inflow in million m³.

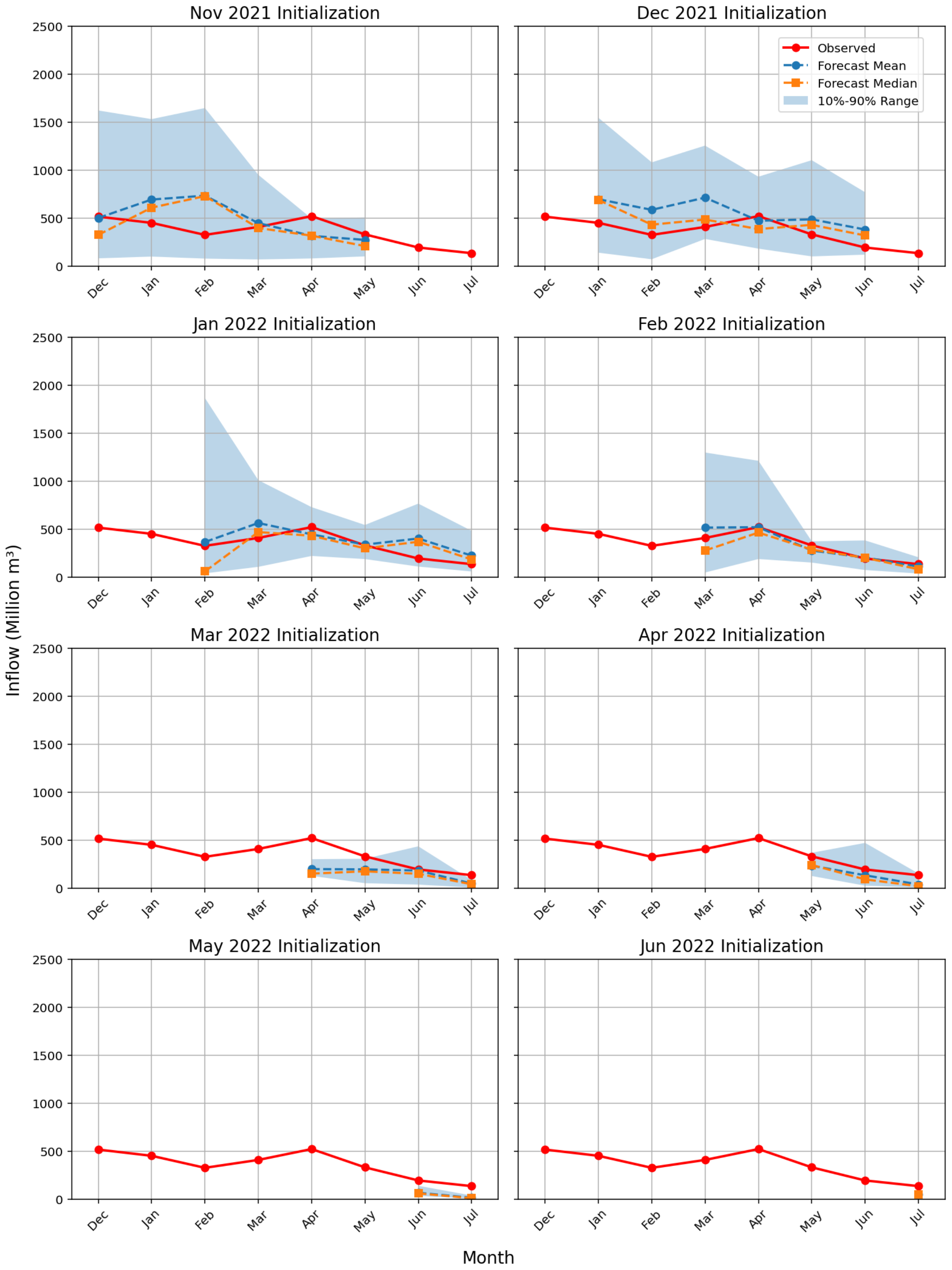

Fig. S 5 Deterministic seasonal streamflow forecasts to the FTO station for water year 2022 in the UFRB. Each subplot corresponds to a different forecast initialization month (Nov–Dec 2021; Jan–Jun 2022) and displays observed inflows from Dec 2021 through Jul 2022 (red circles), forecast mean (blue circles), forecast median (orange squares), and the 10 %–90 % exceedance range (shaded band). The common x-axis spans Dec through Jul, and the y-axis shows inflow in million m³.

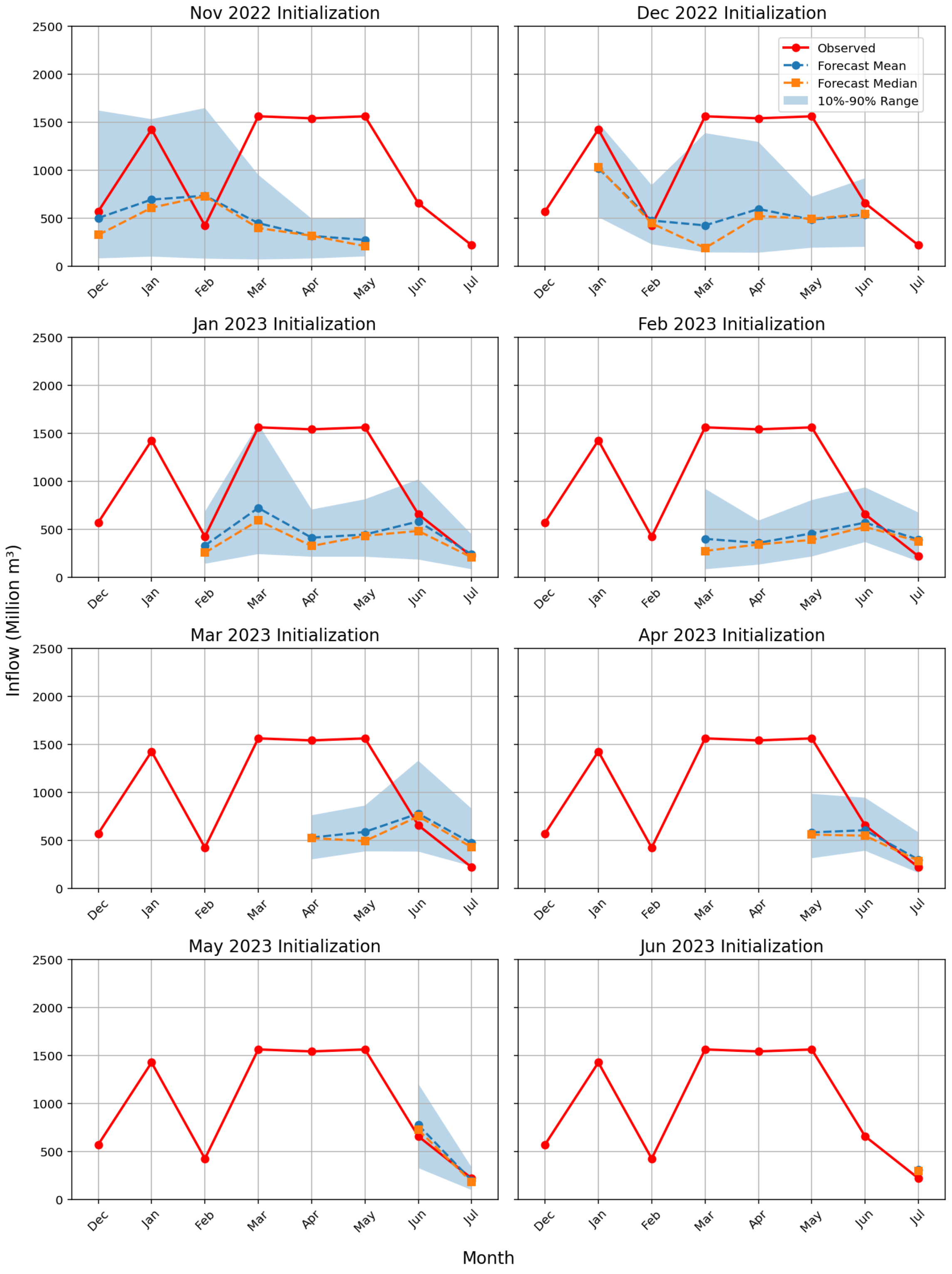

Fig. S 6 Deterministic seasonal streamflow forecasts to the FTO station for water year 2023 in the UFRB. Each subplot corresponds to a different forecast initialization month (Nov–Dec 2022; Jan–Jun 2023) and displays observed inflows from Dec 2022 through Jul 2023 (red circles), forecast mean (blue circles), forecast median (orange squares), and the 10 %–90 % exceedance range (shaded band). The common x-axis spans Dec through Jul, and the y-axis shows inflow in million m³.

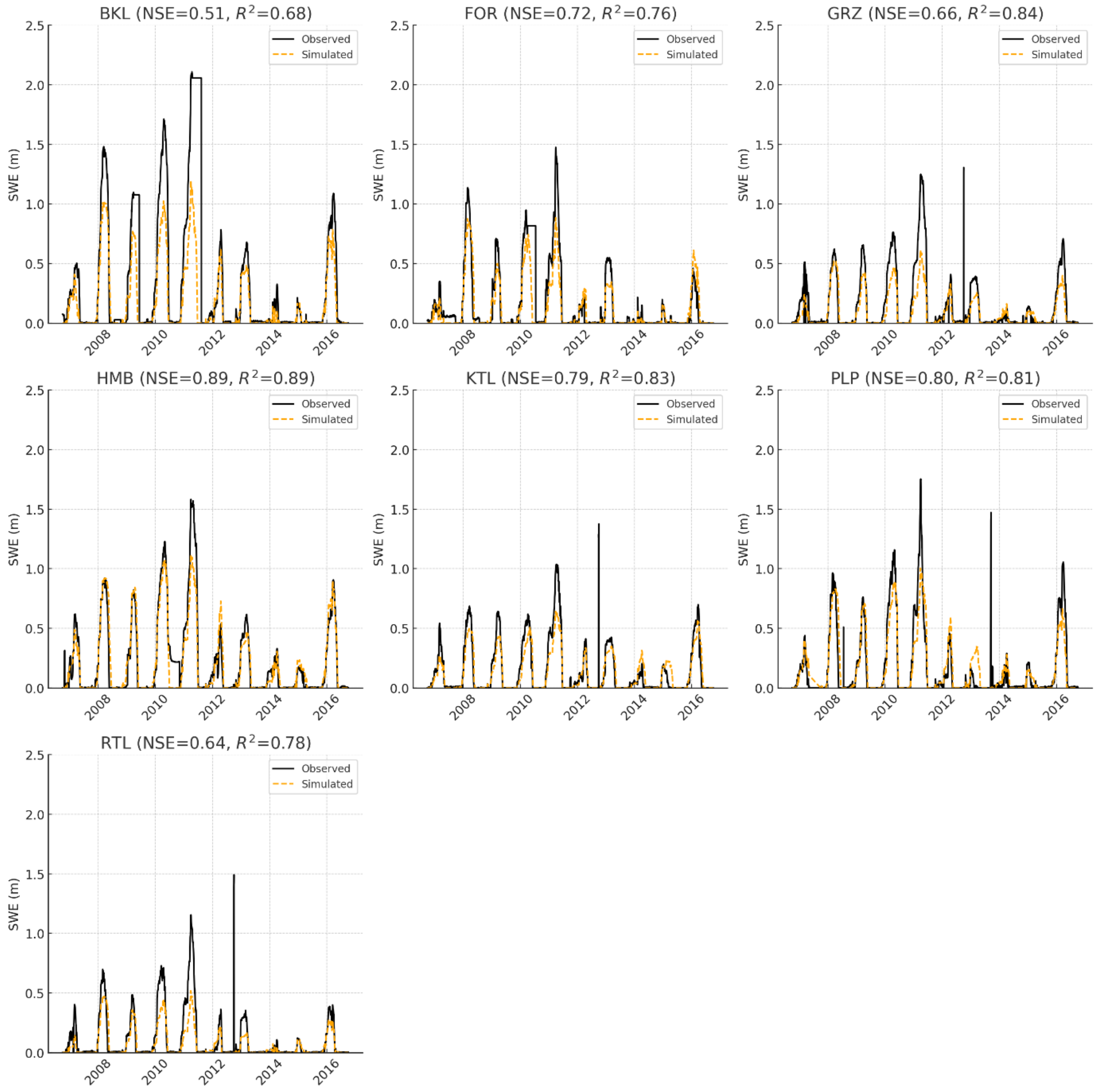

Fig. S 7 Calibration results of the snow model for seven CDEC stations, using daily snow water equivalent (SWE) observation data from 2006 to 2016.

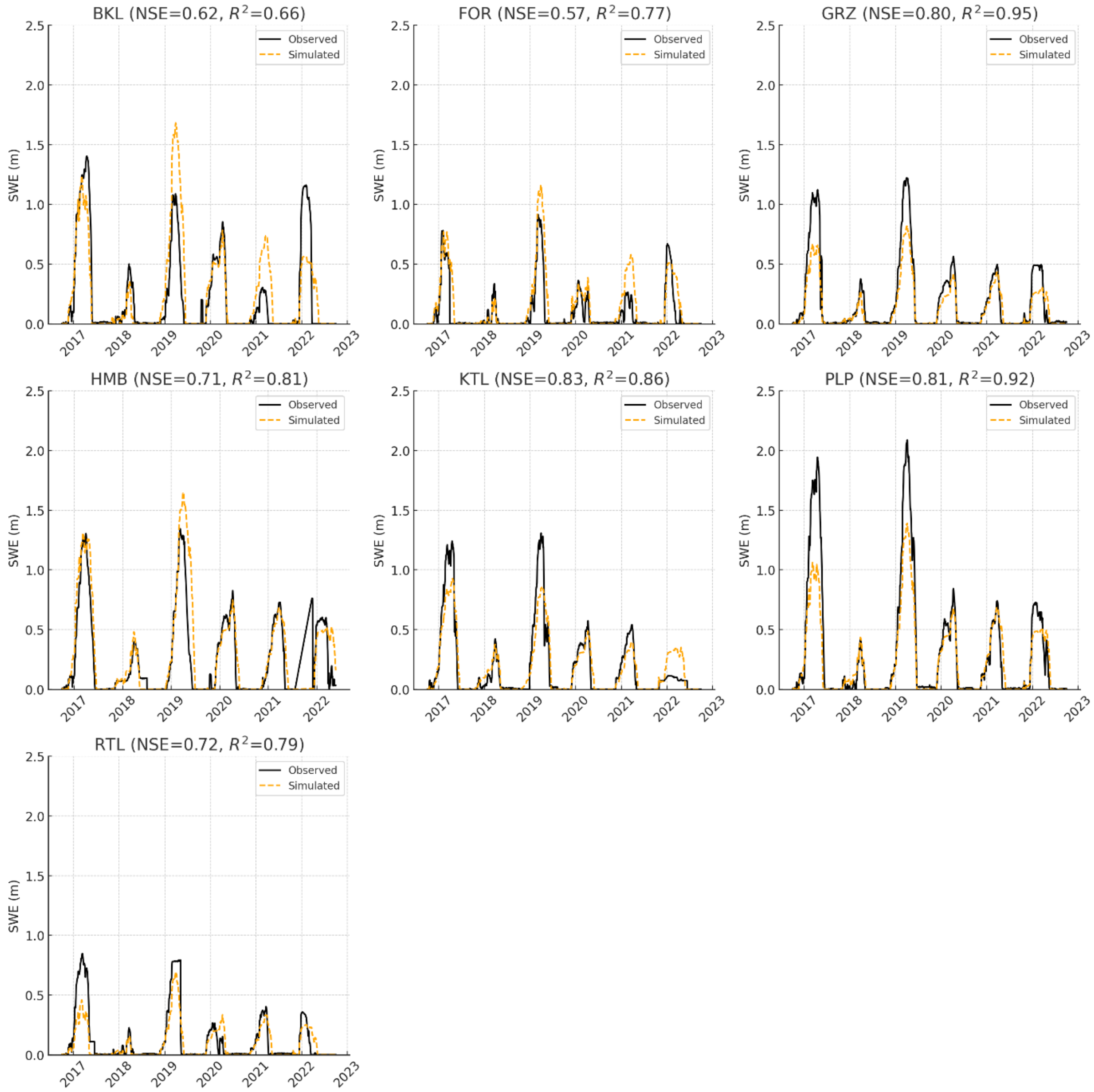

Fig. S 8 Validation results of the snow model for seven CDEC stations, using daily snow water equivalent (SWE) observation data from 2017 to 2022.
