# Supplementary material for: Hybrid physical–statistical framework for seasonal streamflow forecasting in the Upper Feather River Basin, California
Source: Sci Rep. 2025 Aug 30;15:31968. doi: 10.1038/s41598-025-15932-7 (PMC12398547; doi:10.1038/s41598-025-15932-7)
Supplement: Supplementary file 10 — Supplementary material 10 (DOCX 24.9 kb) [file 41598_2025_15932_MOESM10_ESM.docx]

**Supplementary Information Tables**

Table S1 Initialization months and corresponding forecast lead times (in months) along with the forecasted calendar months.

| **Initialization month** | **Lead Times** | | | | | |
| --- | --- | --- | --- | --- | --- | --- |
|  | **1** | **2** | **3** | **4** | **5** | **6** |
|  | **Forecasted Months** | | | | | |
| **Nov** | Dec | Jan | Feb | Mar | Apr | May |
| **Dec** | Jan | Feb | Mar | Apr | May | Jun |
| **Jan** | Feb | Mar | Apr | May | Jun | Jul |
| **Feb** | Mar | Apr | May | Jun | Jul |  |
| **Mar** | Apr | May | Jun | Jul |  |  |
| **Apr** | May | Jun | Jul |  |  |  |
| **May** | Jun | Jul |  |  |  |  |
| **Jun** | Jul |  |  |  |  |  |

Table S2 RMSE (million m^3^) and PBIAS (%) for each forecast initialization month for the deterministic forecasting system.

|  | **Nov-23 init.** | **Dec-23 init.** | **Jan-24 init.** | **Feb-24 init.** | **Mar-24 init.** | **Apr-24 init.** | **May-24 init.** | **Jun-24 init.** |
| --- | --- | --- | --- | --- | --- | --- | --- | --- |
| **RMSE** | 332.3 | 410.3 | 323.9 | 417.5 | 378.6 | 338.7 | 160.1 | 156.8 |
| **PBIAS** | 12.6 | 42.1 | 33.0 | 34.4 | 26.9 | 49.6 | 66.2 | 90.6 |

Table S3 RMSE (million m^3^) and PBIAS (%) for each forecast initialization month for the SES-corrected forecasts.

|  | **Nov-23 init.** | **Dec-23 init.** | **Jan-24 init.** | **Feb-24 init.** | **Mar-24 init.** | **Apr-24 init.** | **May-24 init.** | **Jun-24 init.** |
| --- | --- | --- | --- | --- | --- | --- | --- | --- |
| **RMSE** | 271.0 | 156.6 | 143.4 | 99.2 | 151.9 | 186.7 | 151.4 | 135.0 |
| **PBIAS** | -10.0 | 8.0 | 8.6 | -5.0 | 24.5 | 30.7 | 58.0 | 78.0 |

Table S4 Key characteristics of the four main tributaries of Lake Oroville.

| **Major Division** | **Area (km²)** | **Percent of Watershed Area** | **Mean Daily Flow (m³/s)^*^** | **Mean Annual Inflow (hm³)** | **Percent of Annual Total** |
| --- | --- | --- | --- | --- | --- |
| West Branch | 429 | 4.6 | 9.9 | 312.6 | 6.6 |
| North Fork | 5582 | 59.8 | 91.5 | 2884.4 | 60.4 |
| Middle Fork | 2996 | 32.1 | 42.5 | 1339.5 | 28.1 |
| South Fork | 327 | 3.5 | 7.4 | 232.2 | 4.9 |
| Total | 9334 | 100 | 151.2 | 4768.7 | 100 |
| *Source: California Department of Water Resources | | | | | |

Table S5 Input datasets used by the WEHY model for the UFRB.

| **Data Type** | **Source** | **Spatial Resolution** |
| --- | --- | --- |
| Digital Elevation Model (DEM) | U.S. Geological Survey (USGS) | 1 arc-second |
| Land Use / Land Cover | FRAP 2006 Statewide Land Use / Land Cover Mosaic (formerly CaSIL 2010) | 30 m |
| Soil | USDA NRCS “Web Soil Survey” (STATSGO2, 2013) | Varies by map unit |
| Leaf Area Index (LAI) | MODIS MOD15A2H (Terra/Aqua, average) | 1 km |

Table S6 Characteristics of snow survey stations used for calibrating the snow model.

| **Station Name** | **STA ID** | **County** | **Elevation (m)** |
| --- | --- | --- | --- |
| Bucks Lake | BKL | Plumas | 1790 |
| Four Tress | FOR | Plumas | 1586 |
| Grizzly Ridge | GRZ | Plumas | 2103 |
| Humbug | HMB | Plumas | 1981 |
| Kettle Rock | KTL | Plumas | 2225 |
| Pilot Peak (DWR) | PLP | Plumas | 2073 |
| Rattlesnake | RTL | Plumas | 1893 |
| Source: California Data Exchange Center (CDEC) | | | |

Table S7 WRF output variables used as input to the snow model.

| **WRF output variable** | **Units** |
| --- | --- |
| Precipitation | mm |
| Surface pressure – top pressure | Mb = hPa |
| Potential temperature | K |
| Mixing ratio at the first layer in WRF | g/kg |
| Wind speed at the first layer in WRF | m/s |
| Geopotential height | m |
| Downward shortwave radiation | W/m^2^ |
| Downward longwave radiation | W/m^2^ |
| Air temperature at the first layer in WRF | K |
| u component of wind velocity at 10 m | m/s |
| v component if wind velocity at 10 m | m/s |
| Mixing ratio at 2 m | g/kg |
| Air temperature at 2 m | ^0^C |
